# Enterohemorrhagic *Escherichia coli* Hemolysin Employs Outer Membrane Vesicles to Target Mitochondria and Cause Endothelial and Epithelial Apoptosis

**DOI:** 10.1371/journal.ppat.1003797

**Published:** 2013-12-12

**Authors:** Martina Bielaszewska, Christian Rüter, Lisa Kunsmann, Lilo Greune, Andreas Bauwens, Wenlan Zhang, Thorsten Kuczius, Kwang Sik Kim, Alexander Mellmann, M. Alexander Schmidt, Helge Karch

**Affiliations:** 1 Institute of Hygiene, University of Münster, Münster, Germany; 2 Institute of Infectiology, Center for Molecular Biology of Inflammation (ZMBE), University of Münster, Münster, Germany; 3 Division of Pediatric Infectious Diseases, Johns Hopkins University School of Medicine, Baltimore, Maryland, United States of America; University of Illinois, United States of America

## Abstract

Enterohemorrhagic *Escherichia coli* (EHEC) strains cause diarrhea and hemolytic uremic syndrome resulting from toxin-mediated microvascular endothelial injury. EHEC hemolysin (EHEC-Hly), a member of the RTX (repeats-in-toxin) family, is an EHEC virulence factor of increasingly recognized importance. The toxin exists as free EHEC-Hly and as EHEC-Hly associated with outer membrane vesicles (OMVs) released by EHEC during growth. Whereas the free toxin is lytic towards human endothelium, the biological effects of the OMV-associated EHEC-Hly on microvascular endothelial and intestinal epithelial cells, which are the major targets during EHEC infection, are unknown. Using microscopic, biochemical, flow cytometry and functional analyses of human brain microvascular endothelial cells (HBMEC) and Caco-2 cells we demonstrate that OMV-associated EHEC-Hly does not lyse the target cells but triggers their apoptosis. The OMV-associated toxin is internalized by HBMEC and Caco-2 cells via dynamin-dependent endocytosis of OMVs and trafficked with OMVs into endo-lysosomal compartments. Upon endosome acidification and subsequent pH drop, EHEC-Hly is separated from OMVs, escapes from the lysosomes, most probably via its pore-forming activity, and targets mitochondria. This results in decrease of the mitochondrial transmembrane potential and translocation of cytochrome c to the cytosol, indicating EHEC-Hly-mediated permeabilization of the mitochondrial membranes. Subsequent activation of caspase-9 and caspase-3 leads to apoptotic cell death as evidenced by DNA fragmentation and chromatin condensation in the intoxicated cells. The ability of OMV-associated EHEC-Hly to trigger the mitochondrial apoptotic pathway in human microvascular endothelial and intestinal epithelial cells indicates a novel mechanism of EHEC-Hly involvement in the pathogenesis of EHEC diseases. The OMV-mediated intracellular delivery represents a newly recognized mechanism for a bacterial toxin to enter host cells in order to target mitochondria.

## Author Summary

During the last 30 years, enterohemorrhagic *Escherichia coli* (EHEC) emerged as worldwide causes of diarrhea and hemolytic uremic syndrome, the most common cause of acute kidney failure in children. EHEC hemolysin (EHEC-Hly) is one of the toxins produced by EHEC during infection that afflict the human host. EHEC-Hly belongs to a large family of toxins, whose members typically kill target cells by inserting themselves into the cell membranes, which results in pore formation and ultimately cell lysis. Here we show that EHEC-Hly associated with outer membrane vesicles (OMVs) secreted by EHEC during growth does not lyse human microvascular endothelial and intestinal epithelial cells, which are the major targets in EHEC-mediated human diseases. Instead, the OMV-associated EHEC-Hly uses the OMVs to enter the cells and acts intracellularly. The toxin separates from its carriers in lysosomes, translocates into mitochondria and triggers apoptotic death of the target cells via the mitochondrial pathway. EHEC-Hly is the first known bacterial toxin, which enters host cells via OMVs in order to attack mitochondria. The apoptotic potential of OMV-associated EHEC-Hly indicates a novel mechanism for this toxin to cause cell death during human EHEC infections.

## Introduction

Enterohemorrhagic *Escherichia coli* (EHEC) are global causes of diarrhea and its severe extra-intestinal complication, hemolytic uremic syndrome (HUS) [1]. HUS, the most common cause of acute renal failure in children, is a thrombotic microangiopathy resulting from microvascular endothelial injury in the kidneys and the brain [1]. EHEC produce a spectrum of virulence factors, which plausibly play a role in the pathogenesis of HUS. In addition to Shiga toxins (Stx), which are the major EHEC virulence factors involved in the microvascular endothelial injury [1], [2], several other EHEC toxins can trigger or contribute to this pathology [3]-[6]. The importance of the contribution of EHEC hemolysin (EHEC-Hly) [7], also designated EHEC toxin (Ehx) [8] is increasingly recognized [6], [9].

EHEC-Hly is a 107 kDa pore-forming cytolysin, which belongs to the RTX (repeats-in-toxin) family [7], [8], [10]. The toxin and its activation and secretion machinery are encoded by the EHEC-*hlyCABD* operon, in which EHEC-*hlyA* is the structural gene for EHEC-Hly. The EHEC-*hlyC* product mediates posttranslational activation of EHEC-Hly, and the EHEC-*hlyB*- and EHEC-*hlyD*-encoded proteins transport EHEC-Hly out of the bacterial cell [7], [11], [12]. The contribution of EHEC-Hly to the pathogenesis of HUS is supported by the ability of the toxin to injure microvascular endothelial cells [6]. Moreover, the pro-inflammatory potential of EHEC-Hly [9], its production by the vast majority of EHEC strains associated with HUS [13], [14], and expression of the toxin during infection as demonstrated by the development of anti-EHEC-Hly antibodies in most HUS patients [7] and by increased EHEC-*hlyA* transcription levels in patients' stools [15] offer additional support of the role of EHEC-Hly in the pathogenesis of human diseases.

By investigating the status of EHEC-Hly in bacterial supernatants, we identified two forms of the toxin: a free, soluble EHEC-Hly, and an EHEC-Hly associated with outer membrane vesicles (OMVs), which are released by EHEC bacteria during growth [16]. Similar to the free toxin, the OMV-associated EHEC-Hly binds to human erythrocytes and causes hemolysis. The association with OMVs significantly increases the stability of the toxin and thus prolongs its hemolytic activity compared to the free, soluble form [16] indicating that the OMV-associated EHEC-Hly is a biologically efficient form of the toxin.

The free EHEC-Hly lyses human microvascular endothelial cells [6], most likely via pore formation in the cell membranes as was demonstrated for this toxin form using artificial lipid bilayers [10]. However, the biological consequences of interactions of OMV-associated EHEC-Hly with human microvascular endothelial cells, a major site of injury during HUS, and intestinal epithelial cells, the first barrier encountered by EHEC during infection, are unknown. Therefore, we investigated EHEC-Hly-harboring OMVs for their ability to bind to these cells, to deliver the toxin intracellularly and to trigger cell injury. Additionally, we determined intracellular trafficking of the toxin and of OMVs and the mechanism resulting in cell injury. We demonstrate that OMV-associated EHEC-Hly is internalized by the host cells via the OMVs, but separates from its carriers and subsequently targets mitochondria, triggering apoptosis via the intrinsic caspase-9-dependent pathway.

## Results

### EHEC-Hly rapidly binds to OMVs in growing bacterial cultures

**Figure 1:**
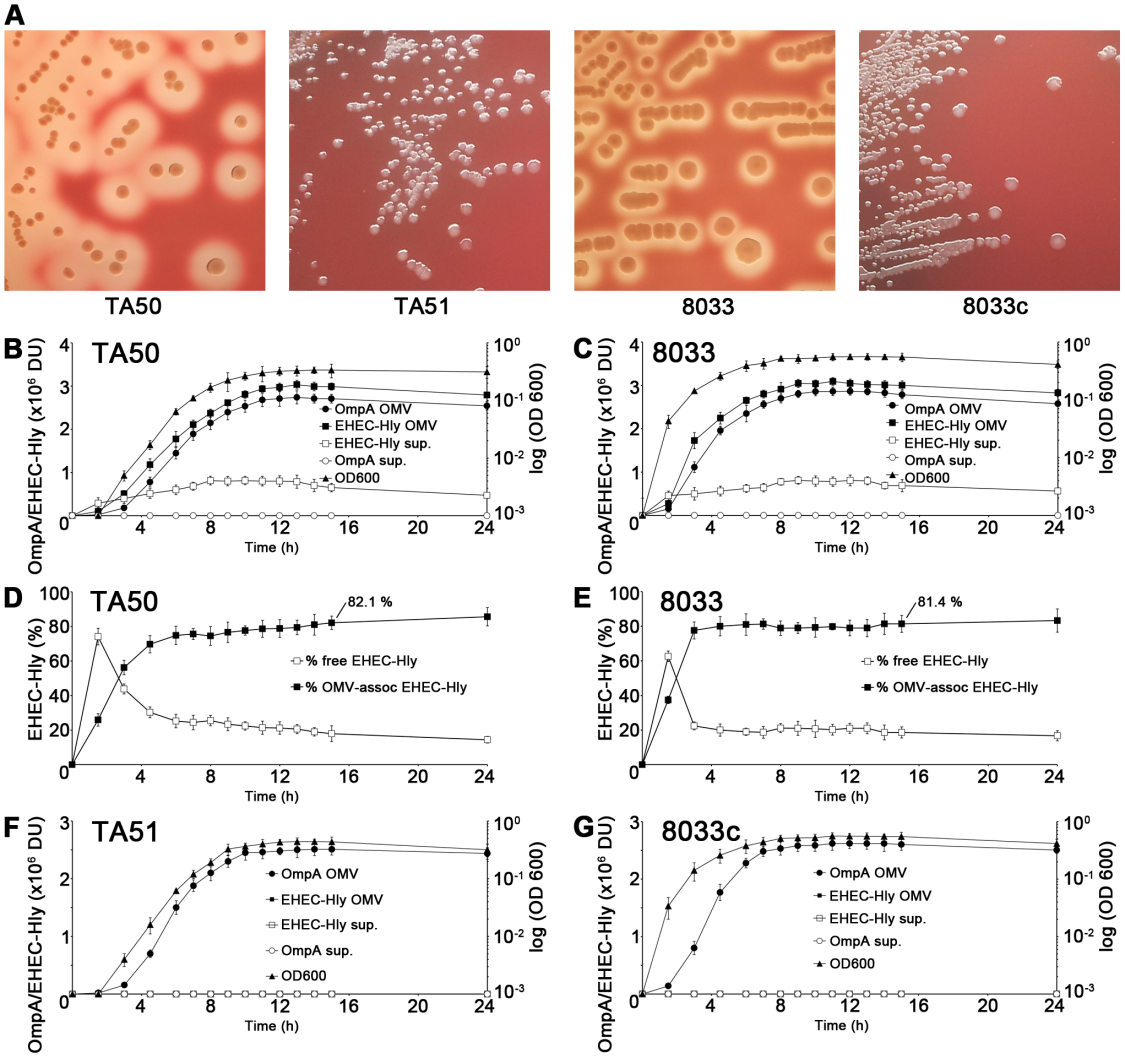
Hemolytic phenotypes and kinetics of EHEC-Hly secretion, OMV production and EHEC-Hly-OMV association in strains used in this study. (A) Strains were grown on enterohemolysin agar for 24 h at 37°C and the plates were examined for hemolysis. Colonies of EHEC-Hly-producing strains TA50 and 8033 are surrounded by zones of hemolysis, whereas no hemolysis is present around colonies of EHEC-Hly-negative strains TA51 and 8033c. The relative contribution of free and OMV-associated EHEC-Hly to hemolysis observed on enterohemolysin agar is unknown. (B, C, F, G) EHEC-Hly-producing strains TA50 (B) and 8033 (C) and EHEC-Hly-negative strains TA51 (F) and 8033c (G) were grown in 1 l of LB broth for 24 h. At each time point of 1.5 h, 3 h, 4.5 h, 6 h to 15 h, and 24 h a 50 ml aliquot of each culture was collected, bacteria were removed by centrifugation and supernatants were sterile filtered. OMVs were pelleted from the filtered supernatants by ultracentrifugation and proteins from supernatants after ultracentrifugation were precipitated with 10% TCA. After resuspending in 100 µl of 20 mM TRIS-HCl, aliquots (9 µl) of the OMV fractions and TCA-precipitated supernatants were separated by SDS-PAGE, immunoblotted with anti-EHEC-Hly or anti-OmpA antibody, and signals were quantified densitometrically and expressed in arbitrary densitometric units (DU). Bacterial growth was monitored by measuring the optical density of the cultures at 600 nm (OD_600_) at each time. (D, E) The percentage of EHEC-Hly associated with OMVs in strains TA50 (D) and 8033 (E) at each time interval was calculated as the percentage of the densitometric signal of OMV-associated EHEC-Hly from the total EHEC-Hly signal (i.e. the sum of OMV-associated and free EHEC-Hly signals). Data in panels B-G are expressed as means ± standard deviations from three independent experiments.

EHEC-Hly is secreted from bacterial cells via type 1 secretion system [8], independently of OMVs, and rapidly binds to OMVs due to its high affinity to these structures [16]. To determine the kinetics of EHEC-Hly secretion, OMV production and EHEC-Hly-OMV association in strains TA50 (*E. coli* K-12 MC1061 harboring EHEC-*hlyCABD* operon from *E. coli* O26:H11) and 8033 (*E. coli* O103:H2 patient isolate) that both produce biologically active EHEC-Hly (Figure 1A), the strains were grown in Luria-Bertani (LB) broth. At defined time intervals OMVs were collected by ultracentrifugation and the OMV fractions (pellets) and resulting supernatants were analyzed for the presence of OMVs and EHEC-Hly using immunoblotting with antibodies against outer membrane protein OmpA (a common component of OMVs of Gram-negative bacteria [17]-[19]) and EHEC-Hly, respectively, followed by densitomeric quantification of the signals. In both strains, the OmpA and EHEC-Hly signals in OMV fractions increased in parallel with bacterial growth, most rapidly during logarithmic phase; in contrast, EHEC-Hly signals in OMV-free supernatants (free EHEC-Hly) remained low throughout the 24 h incubation period (Figure 1B, 1C). This indicated that EHEC-Hly secreted by strains TA50 and 8033 rapidly binds to OMVs released by growing bacteria. The percentage of OMV-associated EHEC-Hly (from the total amount of the toxin present at each time) progressively increased up to 4.5 h of incubation in strain TA50 (72% of total EHEC-Hly OMV-associated) and up to 3 h of incubation in strain 8033 (78% of total EHEC-Hly OMV-associated), and then remained relatively unchanged until 24 h (Figure 1D, 1E). In 15 h cultures of strains TA50 and 8033, from which OMVs used in this study were isolated, 82.1% and 81.4%, respectively, of the total EHEC-Hly was associated with OMVs (Figure 1D, 1E). Quantitative analyses of these OMVs revealed that they contained 3.03±0.8 µg (TA50) and 3.01±0.6 µg (8033) of EHEC-Hly per ml of OMV preparation, corresponding to 5.1±1.2 µg and 4.9±1.1 µg, respectively, of EHEC-Hly per mg of total OMV protein (Figure S1A). Altogether, these experiments demonstrated that the majority of EHEC-Hly secreted by strains TA50 and 8033 rapidly binds to OMVs and that the OMV-associated EHEC-Hly is thus the major form of the toxin in these strains. OMVs, but not EHEC-Hly were present in OMV fractions prepared from control non-hemolytic (Figure 1A) strains TA51 (TA50 vector control) and 8033c (EHEC-*hlyA*-negative derivative of 8033), and none of these strains produced free EHEC-Hly (Figure 1F, 1G).

### Characterization of EHEC-Hly-containing OMVs

To determine whether or not EHEC-Hly is tightly associated with TA50 and 8033 OMVs, we first performed density gradient fractionation of the OMV preparations and analyzed the fractions for the presence of OmpA and EHEC-Hly using immunoblotting. EHEC-Hly was detected in the same OMV density gradient fractions, which also contain OmpA (Figure S1B), suggesting its tight association with OMVs. To confirm this observation, TA50 and 8033 OMVs were subjected to a dissociation assay (see Materials and Methods). Treatment of the OMVs with salt (1 M NaCl), alkali (0.1 M Na_2_CO_3_) or chaotropic reagent (0.8 M urea) did not release EHEC-Hly from OMVs, as demonstrated by the toxin recovery in OMV pellets after ultracentrifugation (Figure S1C). In contrast, exposure of the OMVs to 1% SDS, which totally disrupts membranes, released EHEC-Hly from the pellet to the supernatant (Figure S1C). These experiments demonstrated that EHEC-Hly is tightly associated with TA50 and 8033 OMVs.

**Figure 2:**
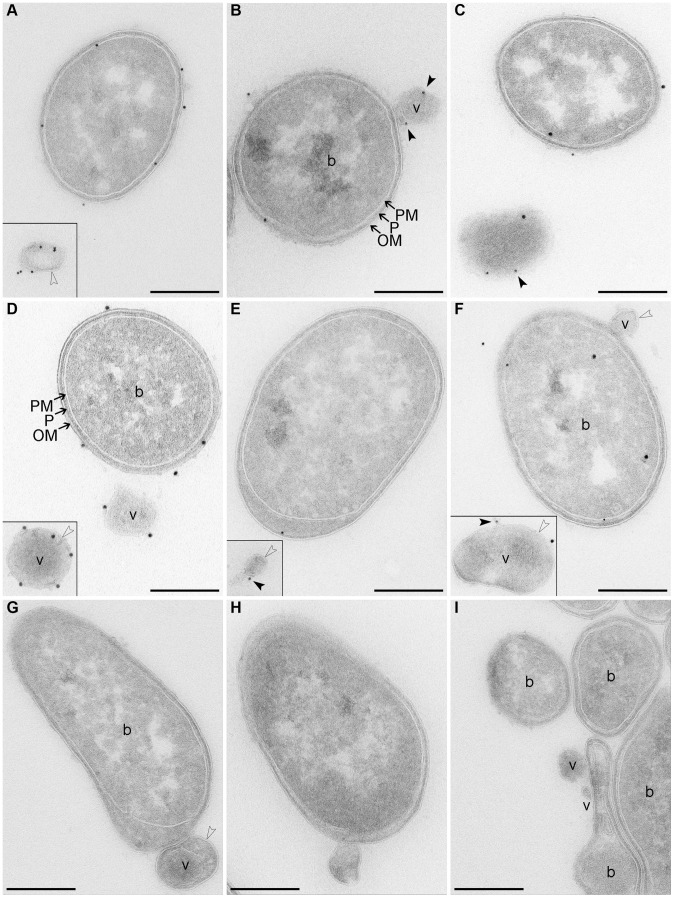
Electron microscopy of OMV-producing bacterial cultures. (**A–F**) Immunogold staining of ultrathin frozen sections of overnight LB agar cultures of strains TA50 (**A–C**) and 8033 (**D–F**) using anti-*E. coli* LPS antibody (recognizing all *E. coli* LPS types) (**A**) or anti-O103 LPS antibody (**D**), anti-EHEC-Hly antibody (**B, E**), or anti-EHEC-Hly antibody and either anti-*E. coli* LPS antibody (**C**) or anti-O103 LPS antibody (**F**); primary antibodies were detected using Protein A Gold with gold particles of 15 nm (anti-LPS antibodies) or 10 nm diameter (anti-EHEC-Hly antibody). (**G, H**) Ultrathin frozen sections of TA50 (**G**) and 8033 (**H**) overnight LB agar cultures stained with Protein A Gold alone, without anti-LPS (**G**) or anti-EHEC-Hly (**H**) antibody. (**I**) Ultrathin frozen section of overnight LB agar culture of EHEC-Hly-negative strain TA51 stained with anti-EHEC-Hly antibody and Protein A Gold with gold particles of 10 nm. Samples were analyzed using a FEI-Tecnai 12 electron microscope. Examples of bacterial cells (b) and OMVs (v) are indicated. Black frames delineate OMVs that were located at longer distances from the OMV-producing bacteria and, therefore, were detected in different microscopic fields. Arrows indicate bacterial outer membrane (OM), periplasmic space (P), and plasma (inner) membrane (PM). Empty arrow heads depict a membrane bilayer surrounding OMVs and black arrow heads depict EHEC-Hly located on OMV surface. Scale bars are 300 nm. Note that using electron microscopy of immunostained ultrathin cryo-sections only 10% - 15% of the total antigen present in the section can be detected [76] explaining relatively low numbers of LPS and EHEC-Hly signals observed.

To gain insight into biochemical composition of EHEC-Hly-containing OMVs, we characterized OMVs from strains TA50 and 8033 for the presence of the outer membrane, periplasmic, inner membrane and cytoplasmic components of bacterial cells, as well as for DNA. Immunoblot analyses using antibodies against marker proteins of the respective compartments demonstrated that OMVs from each strain contain the outer membrane and periplasmic proteins, but not proteins of the inner membrane and the cytoplasm (Figure S1D). Presence of lipopolysaccharide (LPS) of the bacterial outer membrane as a component of TA50 and 8033 OMV membrane was shown using electron microscopy and immunogold staining with antibodies against the respective bacterial LPSs (Figure 2A, 2D). Analysis of OMVs for DNA using Pico green assay revealed DNA in intact OMVs, both untreated and DNase-treated, and in OMVs that were lysed after the DNase treatment (Table S1). This suggests that the DNA is located inside OMVs where it is protected against DNase degradation. No evidence of EHEC-Hly-encoding gene (EHEC-*hlyA*) was found in OMV-associated DNA using PCR (Table S1). The nature of this DNA is yet unknown.

Electron microscopy of ultrathin cryo-sections of TA50 and 8033 LB agar cultures demonstrated blebbing of OMVs from the surface of growing bacteria (Figure 2B, 2F–2H) as well as free OMVs that had already been released from bacterial cells (Figure 2A, 2C–2F). The OMVs are surrounded with a membrane bilayer (Figure 2A, 2D–2H), which - like the bacterial outer membrane - immunoreacts with an antibody against the respective bacterial LPS (Figure 2A, 2D) indicating that the OMV membrane has been derived from the bacterial outer membrane. Staining with anti-EHEC-Hly antibody visualized the toxin on the surface of budding or already released OMVs (Figure 2B, 2E). Double staining with anti-EHEC-Hly and anti-LPS antibodies confirmed an association of the toxin with the OMV membrane (Figure 2C, 2F). No immunogold signals were present in sections of TA50 and 8033 cultures stained only with Protein A Gold in the absence of anti-LPS (Figure 2G) or anti-EHEC-Hly (Figure 2H) antibody, and no EHEC-Hly signals were found in a section of LB agar culture of non-hemolytic strain TA51 stained with anti-EHEC-Hly antibody and Protein A Gold (Figure 2I) demonstrating the specificity of the immunogold staining.

### EHEC-Hly-containing OMVs cause hemolysis but fail to lyse human microvascular endothelial and intestinal epithelial cells

Biological activity of OMV-associated EHEC-Hly was verified by the ability of TA50 and 8033 OMVs to cause hemolysis. The hemolysis appeared after 20 h of incubation and steadily increased up to 32 to 36 h, when it reached a plateau (Figure S1E). However, the TA50 and 8033 OMVs used in the hemolytic dose (300 ng of OMV-associated EHEC-Hly) did not lyse human brain microvascular endothelial cells (HBMEC) and intestinal epithelial cells (Caco-2) even after 48 h of incubation as no release of lactate dehydrogenase (LDH) could be observed (Figure S1E). The lack of the lytic potential of OMV-associated EHEC-Hly towards HBMEC and Caco-2 cells prompted us to investigate other effects of the OMV-associated toxin on these cells.

### EHEC-Hly-containing OMVs bind to and are internalized by HBMEC and Caco-2 cells

**Figure 3:**
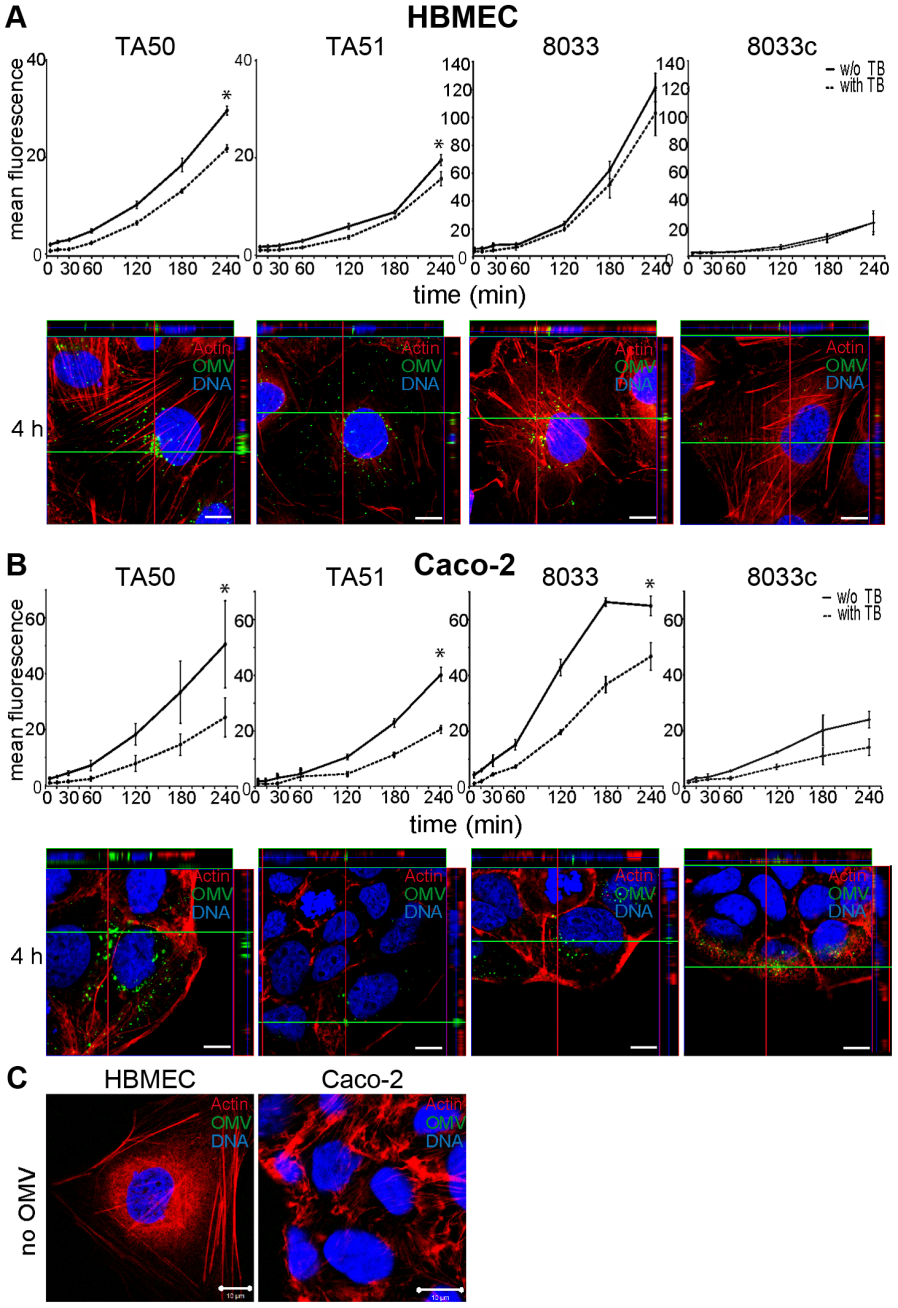
EHEC-Hly-containing and EHEC-Hly-free OMVs bind to and are internalized by HBMEC and Caco-2 cells. (**A**) HBMEC and (**B**) Caco-2 cells were incubated with DiO-labeled OMVs from strains TA50, TA51, 8033 or 8033c for the times indicated and fluorescence was measured using a flow cytometer before (total cell-associated OMVs) and after (internalized OMVs) trypan blue quenching. Data are expressed as geometric means of fluorescence intensities from 10,000 cells after subtraction of background fluorescence of cells without OMVs, and are presented as means ± standard deviations from three independent experiments; * significant difference between cell-bound and internalized OMVs after 4 h (*p*<0.05). In addition, cells incubated with OMVs were analyzed using CLSM. OMVs (green) were detected using rabbit anti-*E. coli* LPS antibody and Alexa Fluor 488-conjugated goat anti-rabbit IgG, actin (red) was counterstained with phalloidin-TRITC and nuclei (blue) with DRAQ5. (**C**) HBMEC and Caco-2 cells were incubated for 4 h with OMV buffer (20 mM TRIS-HCl, pH 8.0) instead of OMVs and analyzed using CLSM as described above. Pictures were taken using a laser-scanning microscope (LSM 510 META microscope, equipped with a Plan-Apochromat 63x/1.4 oil immersion objective). All three fluorescence images were merged and confocal Z-stack projections are included in all images in panels A and B. The cross hairs show the position of the xy and yz planes. Scale bars are 10 µm.

To determine if EHEC-Hly-containing OMVs associate with HBMEC and Caco-2 cells, the cells were incubated with OMVs labeled with the fluorescent membrane dye 3,3 dioctadecyloxacarbocyanine perchlorate (DiO) and the kinetics of OMV uptake was monitored using flow cytometry (Figure 3A, 3B). To distinguish internalized from cell-bound but non-internalized OMVs, extracellular DiO-OMV fluorescence was quenched with trypan blue. DiO-labeled TA50 and 8033 OMVs bound to each cell line within 5 min of incubation, and the OMV-cell associations steadily increased up to 3 to 4 h (Figures 3A, 3B). The proportions of trypan blue non-quenched TA50 and 8033 OMVs also steadily increased during time (Figure 3A, 3B) and reached 73.6% and 84.4% in HBMEC and 48.1% and 72% in Caco-2 cells, respectively, after 4 h (Table S2). This indicated that large parts of OMVs were internalized after 4 h, even though the proportions of internalized OMVs were, in particular in Caco-2 cells, significantly lower than those of cell-bound OMVs (Figure 3A, 3B). EHEC-Hly-free DiO-labeled OMVs TA51 and 8033c also bound to cellular membranes and were internalized by each cell line in a time-dependent manner, but their cell associations were less than those of the respective EHEC-Hly-harboring OMVs (Figure 3A, 3B). To confirm the OMV internalization, HBMEC and Caco-2 monolayers were incubated with DiO-labeled OMVs for 4 h and native cells were analyzed for fluorescence before and after trypan blue quenching using differential interference contrast (DIC) microscopy and confocal laser scanning microscopy (CLSM). Most of DiO-labeled OMVs were found to be located within cell bodies and these OMVs were not quenched with trypan blue demonstrating that they had been internalized (Figure S2).

Visualization of the OMV interactions with HBMEC and Caco-2 cells using CLSM of fixed and stained cells agreed with the flow cytometry. OMVs from all strains were associated with cellular membranes after 15 min and the OMV cell adherence and internalization increased with time. After 4 h nearly all OMVs were internalized and accumulated in the perinuclear regions (Figure 3A, 3B, panels 4 h). No OMV signals were observed in cells incubated for 4 h with OMV buffer (20 mM TRIS-HCl, pH 8.0) in lieu of OMVs (Figure 3C), and in OMV-treated cells stained with secondary antibody (Alexa Fluor 488-conjugated goat anti-rabbit IgG) in the absence of primary (anti-*E. coli* LPS) antibody (Figure S3A). Taken together, these experiments showed that both EHEC-Hly-harboring OMVs and EHEC-Hly-free OMVs adhered to and were internalized by HBMEC and Caco-2 cells and that EHEC-Hly is not necessary for but might contribute to these processes.

### EHEC-Hly-containing OMVs are internalized via dynamin-dependent endocytosis

**Figure 4:**
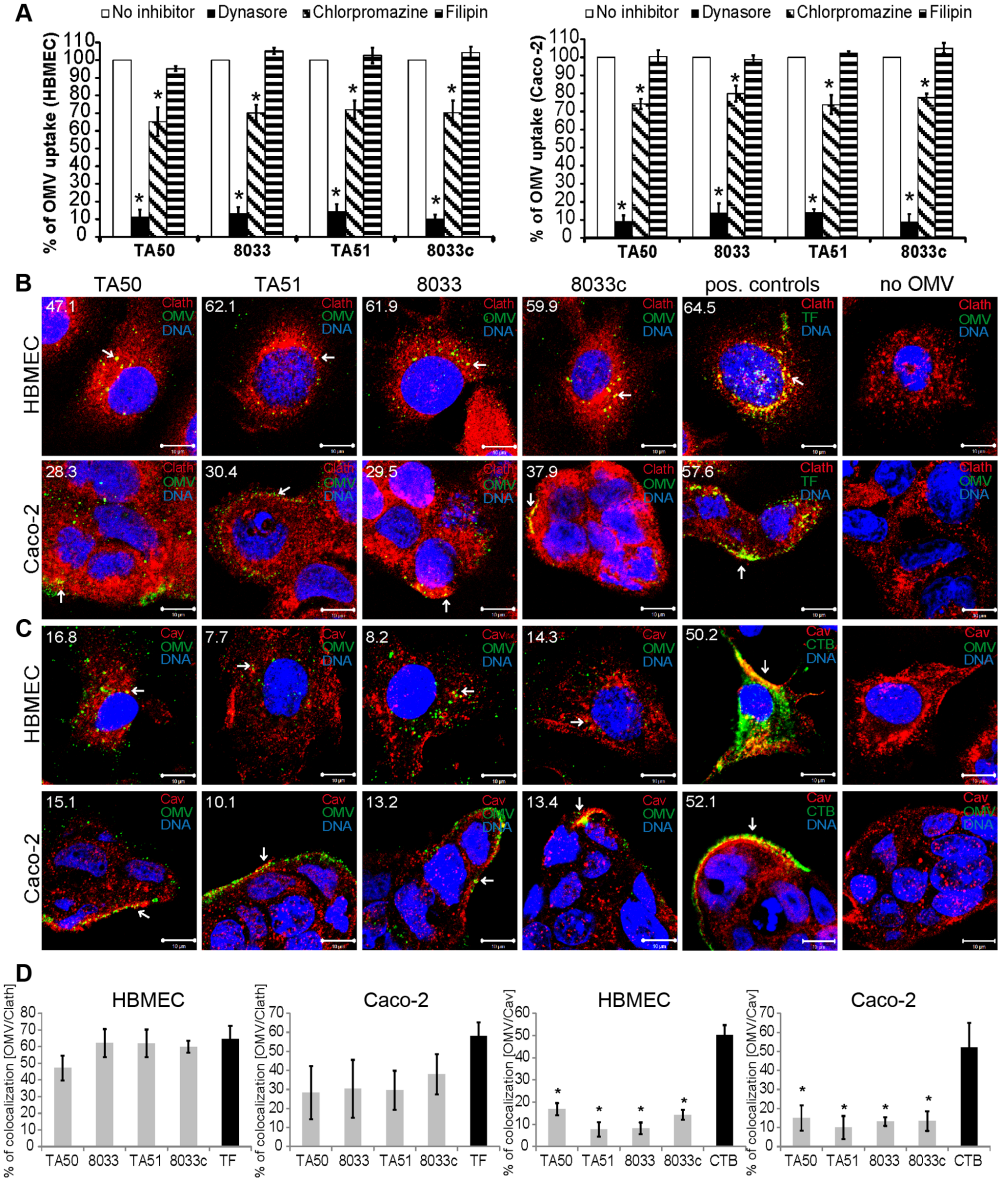
EHEC-Hly-containing OMVs are internalized via dynamin-dependent endocytosis. (**A**) OMVs from strains TA50, 8033, TA51 or 8033c labeled with rhodamine isothiocyanate B-R18 were incubated for 4 h with HBMEC and Caco-2 cells that had been pretreated (1 h) with inhibitors of endocytosis including dynasore (80 µM), chlorpromazine (15 µg/ml), or filipin III (10 µg/ml) or remained inhibitor-untreated (control). Fluorescence was measured using a plate reader and OMV uptake (reflected by fluorescence intensity) in the presence of each inhibitor was expressed as the percentage of OMV uptake by control, inhibitor-untreated cells. * significantly decreased (*p*<0.05) compared to control cells (unpaired Student's *t* test). Data are presented as means ± standard deviations from three independent experiments. (**B, C**). HBMEC and Caco-2 cells were incubated with TA50, TA51, 8033 or 8033c OMVs or with TF-488 or CTB-488 (positive controls) or with 20 mM TRIS-HCl buffer (no OMV) for 10 min and analyzed by CLSM. OMVs were stained with rabbit anti-*E. coli* LPS antibody and Alexa Fluor 488-conjugated goat anti-rabbit IgG (green), and clathrin (**B**) or caveolin (**C**) were stained using the respective mouse monoclonal antibody and Cy3-conjugated goat anti-mouse IgG (red). Nuclei were stained with DRAQ5 (blue). Pictures were taken using a laser-scanning microscope (LSM 510 META microscope equipped with a Plan-Apochromat 63x/1.4 oil immersion objective). All three fluorescence images were merged and consisted of one optical section of a z-series with a pinhole of 1 airy unit. Colocalized red and green signals appear in yellow (examples depicted by arrows). Scale bars are 10 µm. The percentages of colocalizations between OMVs and clathrin or caveolin (and TF-488/CTB-488 and clathrin/caveolin) were calculated using BioImageXD6 colocalization tool and are indicated by white numbers (averages from at least five different samples) in the images and depicted graphically in panel (**D**).

OMVs from bacterial pathogens can enter host cells via lipid raft/caveolae-dependent or clathrin-dependent endocytosis [20], [21]. To characterize the endocytic pathway utilized by EHEC-Hly-containing OMVs, we first determined effects of various inhibitors of endocytosis on uptake of rhodamine isothiocyanate B-R18-labeled TA50 and 8033 OMVs, as well as EHEC-Hly-free control OMVs (TA51, 8033c) by HBMEC and Caco-2 cells. Dynasore, an inhibitor of dynamin-1, which promotes scission of endocytic vesicles from plasma membrane [22], inhibited the OMV uptake to 10% – 14% and to 9% – 14% of their uptake by untreated HBMEC and Caco-2 cells, respectively (*p*<0.05) (Figure 4A). Significant inhibition of OMV uptake (to 65% – 72% of control in HBMEC and to 74% – 79% of control in Caco-2) (*p*<0.05) was also caused by chlorpromazine, an inhibitor of clathrin-mediated endocytosis [23] (Figure 4A). In contrast, filipin, a cholesterol-sequestering agent that disrupts lipid rafts and caveolae [24] had no significant inhibitory effect on OMV uptake (Figure 4A). These experiments indicated that endocytosis of EHEC-Hly-containing and EHEC-Hly-free OMVs is dynamin-dependent and might be clathrin-mediated.

To further explore relative contribution of clathrin versus caveolin to OMV endocytosis and to gain insight into the role of EHEC-Hly in this process, HBMEC and Caco-2 cells were incubated with EHEC-Hly-containing (TA50, 8033) and EHEC-Hly-free (TA51, 8033c) OMVs for 10 min and colocalization of OMVs with clathrin and caveolin was determined using CLSM. In HBMEC, colocalization rates of OMVs with clathrin ranged from 47.1% to 62.1% and were similar to that of Alexa Fluor 488-conjugated transferrin (TF-488) (64.5%), a well-established marker for clathrin-mediated endocytosis [25] (Figure 4B, 4D). In Caco-2 cells, colocalization rates of OMVs with clathrin were between 28.3% and 37.9% (Figure 4B), being lower, but not significantly, than that of TF-488 (57.6%) (Figure 4B, 4D). In contrast to clathrin, colocalization of OMVs with caveolin (7.7% – 16.8% in HBMEC and 10.1% – 15.1% in Caco-2 cells) was in each cell line significantly lower than that of Alexa Fluor 488-conjugated cholera toxin B subunit (CTB-488) (50.2% and 52.1%, respectively), a marker for caveolin-mediated endocytosis [24] (Figure 4C, 4D). No colocalization signals with clathrin or caveolin were observed in cells treated with 20 mM TRIS-HCl in lieu of OMVs (Figure 4B, 4C). No significant differences were observed in any cell line between clathrin colocalization rates of EHEC-Hly-containing (TA50, 8033) and EHEC-Hly-free (TA51, 8033c) OMVs (Figure 4D) indicating that the clathrin-mediated endocytosis of OMVs does not require EHEC-Hly. In summary, these experiments showed that EHEC-Hly-containing OMVs are internalized by HBMEC and Caco-2 cells via dynamin-dependent and largely clathrin-mediated endocytosis and that the OMV endocytosis occurs independently of the presence of EHEC-Hly.

### EHEC-Hly is internalized via OMV endocytosis and subsequently translocates into mitochondria

**Figure 5:**
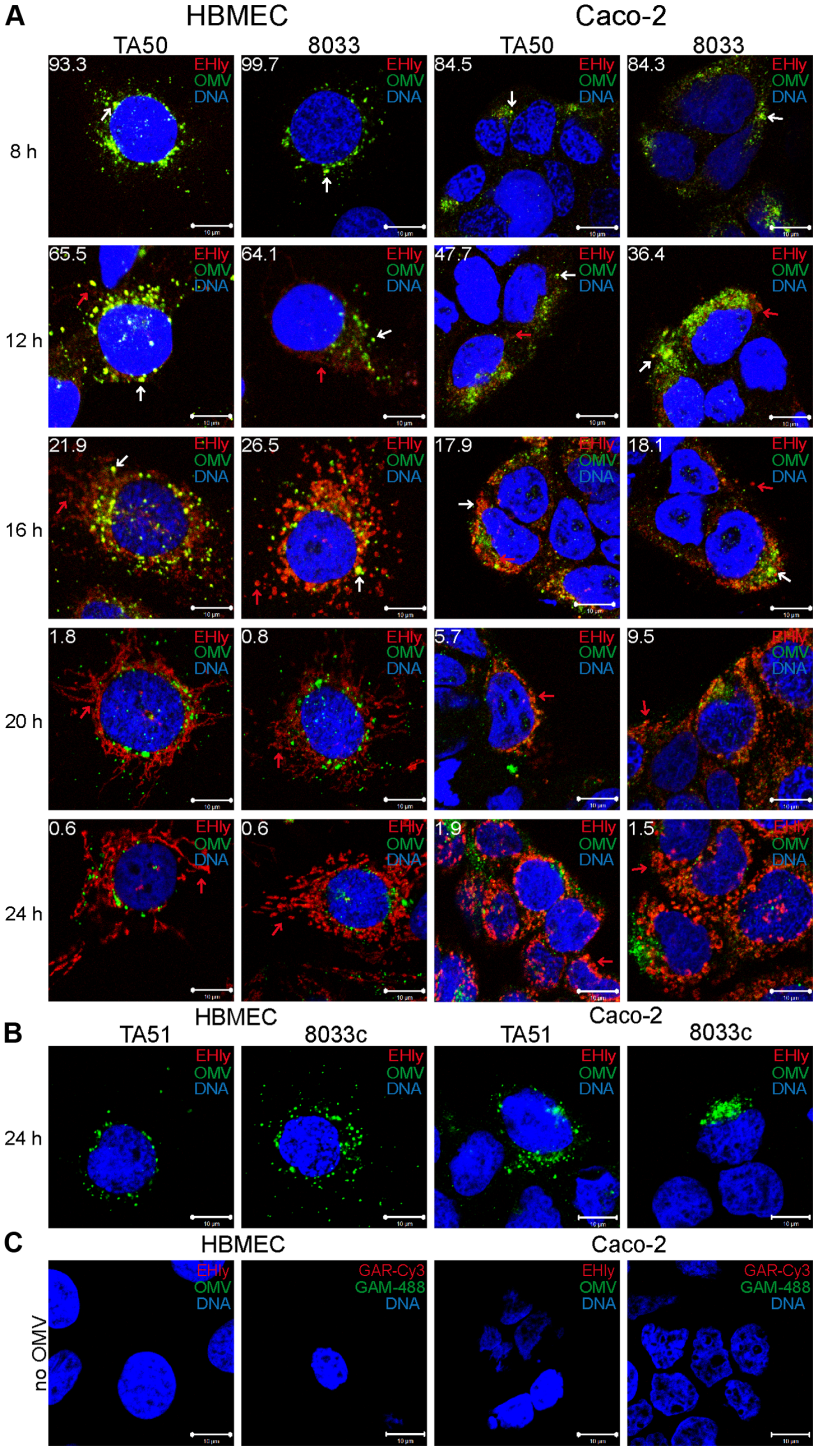
EHEC-Hly separates from OMVs during intracellular trafficking. (**A**) HBMEC and Caco-2 cells were incubated with TA50 or 8033 OMVs for the times indicated and analyzed using CLSM. OMVs were stained using mouse anti-*E. coli* LPS antibody and Alexa Fluor 488-conjugated goat anti-mouse IgG (green), and EHEC-Hly (EHly) was stained using rabbit anti-EHEC-Hly antibody and Cy3-conjugated goat anti-rabbit IgG (red). Nuclei were stained with DRAQ5 (blue). Pictures were taken and processed as described in the legend to Figure 4. Colocalized red and green signals appear in yellow (examples are indicated by white arrows). Red arrows indicate examples of red signal of EHEC-Hly dissociating from OMVs during time. The percentages of colocalization between OMVs and EHEC-Hly were calculated using BioImageXD6 colocalization tool and are indicated by white numbers (averages from at least five different samples). (**B**) HBMEC and Caco-2 cells were incubated for 24 h with EHEC-Hly-free OMVs from strains TA51 or 8033c and stained as described in panel A. (**C**) HBMEC and Caco-2 cells were incubated for 24 h with 20 mM TRIS-HCl (OMV buffer) instead of OMVs and stained for OMVs and EHEC-Hly as described in panel A or stained with secondary antibodies in the absence of primary antibodies. Scale bars in all panels are 10 µm.

**Figure 6:**
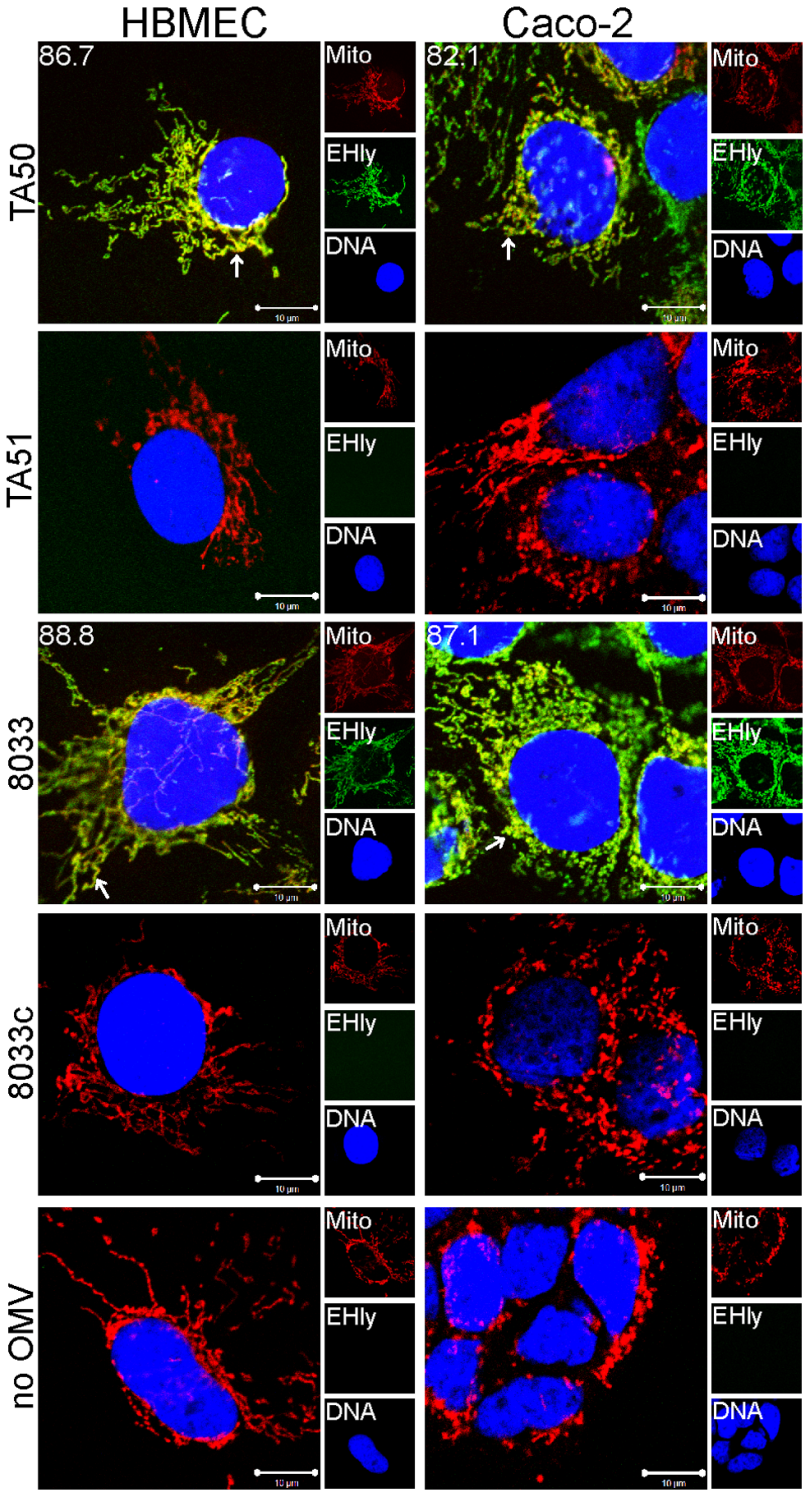
EHEC-Hly colocalizes with mitochondria. HBMEC and Caco-2 cells were incubated with EHEC-Hly-containing OMVs from strains TA50 and 8033 or with EHEC-Hly-free OMVs from strains TA51 and 8033c (controls) or with 20 mM TRIS-HCl buffer in lieu of OMVs for 24 h. EHEC-Hly (EHly) was stained with anti-EHEC-Hly antibody and Alexa Fluor 488-conjugated goat anti-rabbit IgG (green) and mitochondria (Mito) were stained with MitoTracker Orange CMTMRos (red). DNA was stained with DRAQ5 (blue). Pictures were taken using a laser-scanning microscope (LSM 510 META microscope, equipped with a Plan-Apochromat 63x/1.4 oil immersion objective). All three fluorescence images were merged (left panels; colocalized red and green signals appear in yellow and examples are depicted by arrows) and single fluorescence channels are shown in the right panels. Pictures consisted of one optical section of a z-series with a pinhole of 1 airy unit. Scale bars are 10 µm. Note that mitotracker signals in cells treated with EHEC-Hly-containing OMVs (TA50, 8033) are slightly diffuse compared to those in cells treated with EHEC-Hly-free OMVs (TA51, 8033c) and in OMV-untreated cells, likely because of reduction of the mitochondrial transmembrane potential induced by EHEC-Hly at this time (see Figure 11E, 11F).

To assess if EHEC-Hly was internalized with OMVs and to monitor its association with OMVs during intracellular trafficking, cells were incubated with TA50 or 8033 OMVs for 1 h to 24 h and analyzed by CLSM. Association of the toxin with OMVs was quantified by calculating colocalization rates of OMV and EHEC-Hly signals at each time. In both HBMEC and Caco-2 cells, EHEC-Hly colocalized with OMVs up to 8 h of incubation. At this time point the colocalization rates of OMVs and EHEC-Hly reached 93.3% – 99.7% in HBMEC and 84.3% – 84.5% in Caco-2 cells and the OMV/EHEC-Hly complexes were localized predominantly in perinuclear regions (Figure 5A, panel 8 h, Figure S4A). After 12 h, EHEC-Hly remained partially associated with OMVs (64.1% – 65.5% colocalization in HBMEC and 36.4% – 47.7% colocalization in Caco-2 cells), but the toxin began to be dissociated from OMVs as demonstrated by its appearance also outside the OMVs (Figure 5A, panel 12 h, Figure S4A). The separation of EHEC-Hly from OMVs continued until 16 h (21.9% – 26.5% colocalization in HBMEC and 17.9% – 18.1% colocalization in Caco-2 cells), when most of the toxin was located outside OMVs in the form of granular and tubular patches (Figure 5A, panel 16 h, Figure S4A). After 20 h to 24 h the toxin was separated entirely from the perinuclear OMVs (0.6% colocalization in HBMEC and 1.5% – 1.9% colocalization in Caco-2 cells after 24 h) and accumulated in a cell compartment resembling mitochondria (Figure 5A, panels 20 h and 24 h, Figure S4A). The presence of EHEC-Hly in mitochondria was confirmed by strong (>80%) colocalization of the toxin with mitotracker, a fluorescent dye that specifically stains mitochondria [26] (Figure 6). No EHEC-Hly, but only OMVs were detected in cells incubated for 24 h with EHEC-Hly-free OMVs from strains TA51 and 8033c (Figure 5B). Neither OMVs nor EHEC-Hly were found in control cells treated with 20 mM TRIS-HCl instead of OMVs (Figure 5C) and in OMV-treated cells stained with secondary antibodies in the absence of primary antibodies (Figure S3B); this confirmed the specificity of the signals observed in cells treated with EHEC-Hly-containing OMVs.

To confirm that the intracellular EHEC-Hly signals detected in cells treated with EHEC-Hly-containing OMVs (Figure 5A, panels 8 h to 24 h) represent indeed only the OMV-delivered toxin, we incubated in a control experiment HBMEC and Caco-2 cells for 24 h with a sublytic dose of free recombinant EHEC-Hly prepared from strain TA50. In contrast to OMV-delivered EHEC-Hly, which was at this time already located in mitochondria (Figure 5A, panel 24 h, Figure 6), free EHEC-Hly remained restricted to the cell surface up to 24 h without entering cells (Figure S4B). This confirmed that EHEC-Hly detected intracellularly in HBMEC and Caco-2 cells exposed to EHEC-Hly-containing OMVs originates solely from the OMV-delivered toxin. The apparent increase of EHEC-Hly intracellular signals during time (Figure 5A, panels 8 h to 24 h) plausibly results from a gradual dissociation of EHEC-Hly from OMVs entering continually cells and accumulation of the toxin in mitochondria. Importantly, this experiment clearly showed that association of EHEC-Hly with OMVs is a prerequisite for its internalization by host cells and that OMVs serve as vehicles for the intracellular toxin delivery. Taken together, these data demonstrate that in HBMEC as well as in Caco-2 cells EHEC-Hly is internalized via OMVs and remains associated with the OMVs for at least 8 h. The OMV/EHEC-Hly complexes are trafficked towards perinuclear regions where EHEC-Hly dissociates from OMVs and subsequently targets mitochondria by an OMV-independent process. Because EHEC-Hly was never observed in the endoplasmic reticulum or the trans-Golgi-network (Figure S4C, S4D), we further investigated whether the toxin might be released from OMVs in endo-lysosomal compartments, which also accumulate in perinuclear regions.

### EHEC-Hly is released from OMVs in CD63-positive endo-lysosomal compartments

**Figure 7:**
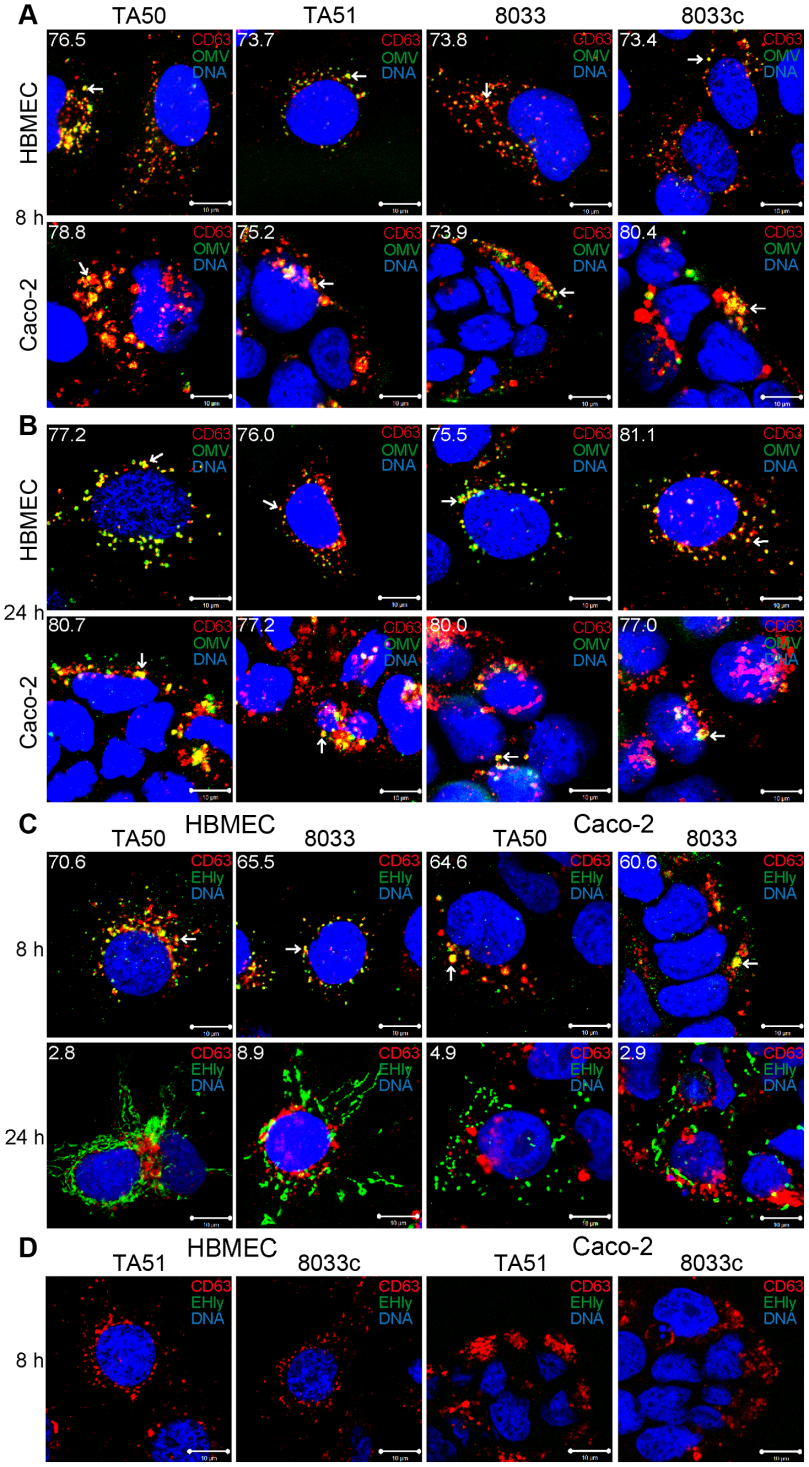
Colocalization of OMVs and EHEC-Hly with endo-lysosomal compartments detected with anti-CD63 antibody. (**A, B**) HBMEC and Caco-2 cells were incubated with EHEC-Hly-containing (TA50 or 8033) or EHEC-Hly-free (TA51 or 8033c) OMVs for 8 h (**A**) and 24 h (**B**). OMVs were stained with rabbit anti-*E. coli* LPS antibody and Alexa Fluor 488-conjugated goat anti-rabbit IgG (green), lysosomes with mouse anti-CD63 antibody and Cy3-conjugated goat anti-mouse IgG (red), and nuclei with DRAQ5 (blue). (**C, D**) HBMEC and Caco-2 cells were incubated with EHEC-Hly-containing (TA50 or 8033) (**C**) or EHEC-Hly-free (TA51 or 8033c) OMVs (**D**) for 8 h and 24 h and stained as described above except that in lieu of OMVs, EHEC-Hly (EHly) was detected with rabbit anti-EHEC-Hly antibody and Alexa Fluor 488-conjugated goat anti-rabbit IgG (green). Pictures were taken and processed as described in the legend to Figure 4. Colocalized red and green signals appear in yellow (examples indicated by arrows). White numbers indicate the percentages of OMVs (**A, B**) and EHEC-Hly (**C**) colocalized with CD63-positive compartments (averages from at least five different samples) calculated using the BioImageXD6 colocalization tool. Scale bars are 10 µm. The images in panel D (8 h of incubation) are also representative of 24 h (no EHEC-Hly was detected in cells treated with EHEC-Hly-free OMVs at any of these time points).

To determine localization of OMVs and EHEC-Hly in endo-lysosomal compartments, we analyzed their colocalizations with the lysosome-associated membrane protein 3 (LAMP3/CD63) after 8 h, 16 h and 24 h of incubation. In all time intervals, EHEC-Hly-positive (TA50 and 8033) and EHEC-Hly-negative (TA51 and 8033c) OMVs strongly (>70%) colocalized with CD63-positive compartments in both HBMEC and Caco-2 cells (Figure 7A, 7B, Figure S5A, S5D). In contrast to OMVs, and in accordance with its time-dependent separation from OMVs (Figure 5A), EHEC-Hly strongly colocalized with CD63-positive compartments after 8 h (65.5% – 70.6% in HBMEC and 60.6% – 64.6% in Caco-2 cells) (Figure 7C, panel 8 h, Figure S5E), but the colocalization significantly decreased after 16 h (24.6% – 26.0% in HBMEC and 25.5% – 27.8% in Caco-2 cells) (Figure S5B, S5E) and 24 h (2.8% – 8.9% in HBMEC and 2.9% – 4.9% in Caco-2 cells) (Figure 7C, panel 24 h, Figure S5E). No EHEC-Hly was detected in cells treated with EHEC-Hly-free OMVs (Figure 7D), and no OMVs and EHEC-Hly were detected in control cells treated with OMV buffer instead of OMVs (Figure S5C), as well as in OMV-treated cells stained with secondary antibodies in the absence of primary antibodies (Figure S3A). Altogether, these data indicated that after its internalization via OMVs, EHEC-Hly trafficks together with OMVs into CD63-positive endo-lysosomal compartments, from where it is subsequently released and translocates to mitochondria, while OMVs remain in the CD63-positive compartments for up to 24 h.

**Figure 8:**
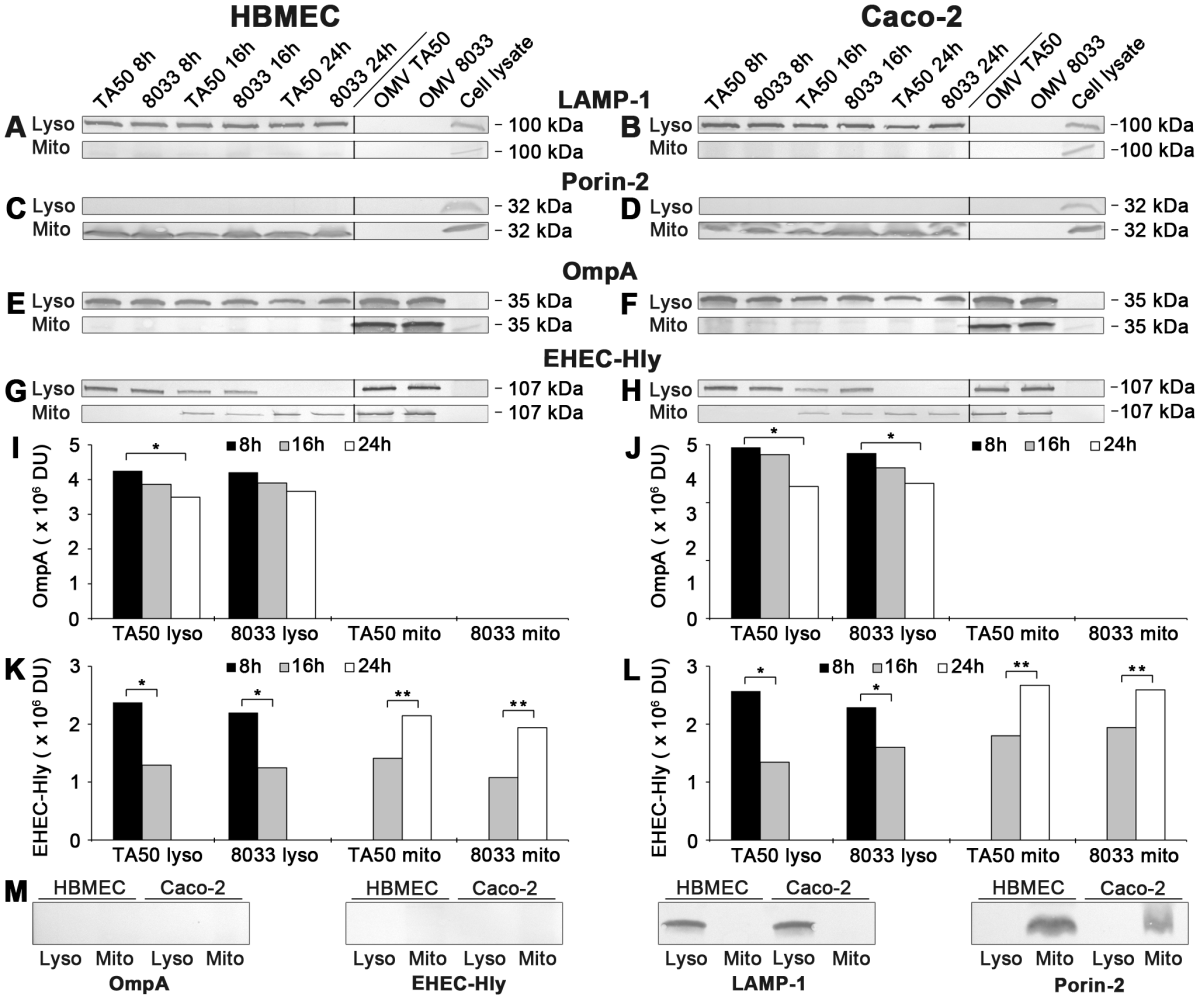
Detection of OMVs and EHEC-Hly in lysosomal and mitochondrial fractions of cells treated with EHEC-Hly-containing OMVs. (A-H) Lysosomes (Lyso) and mitochondria (Mito) were isolated from HBMEC and Caco-2 cells, which had been incubated with OMVs TA50 or 8033 for 8 h, 16 h and 24 h as described in Materials and Methods. The lysosomal and mitochondrial fractions were analyzed using Western blot for marker proteins of each respective fraction including LAMP-1 (A, B) and porin-2 (C, D), and for OMVs (anti-OmpA antibody) (E, F) and EHEC-Hly (G, H). Signals elicited from the lysosomal and mitochondrial fractions at each time are shown in the first part of each immunoblot, whereas the second part (separated by a line) includes controls run at the same gel (isolated OMVs TA50 and 8033 and cell lysates). The sizes of immunoreactive bands are indicated along the right sides of the blots. (I–L) Densitometric quantification of OmpA (I, J) and EHEC-Hly (K, L) signals shown in E, F and G, H, respectively, using Quantity One software; * significantly decreased (*p*<0.05) compared to 8 h; ** significantly increased (*p*<0.05) compared to 16 h (unpaired Student's *t* test). (M) Immunoblots of isolated lysosomal and mitochondrial fractions from control, OMV non-treated HBMEC and Caco-2 cells with the antibodies indicated.

To confirm the CLSM-based data on the trafficking of OMV-EHEC-Hly complexes into endo-lysosomal compartments (Figure 7A, 7C, panels 8 h) and the subsequent separation of EHEC-Hly from OMVs (Figure 5A) and its translocation to mitochondria (Figure 5A, Figure 7C, panel 24 h, Figure S5B), we isolated lysosomes and mitochondria from HBMEC and Caco-2 cells incubated with TA50 or 8033 OMVs for 8 h, 16 h and 24 h, and determined the presence of OMVs and EHEC-Hly in these subcellular compartments using immunoblotting with anti-OmpA and anti-EHEC-Hly antibody, respectively. The identity of the isolated fractions was confirmed by the detection of LAMP-1, a lysosomal marker protein, and porin-2, a mitochondrial marker protein, in the respective fractions (Figure 8A–8D). As demonstrated by OmpA immunoblotting, both TA50 and 8033 OMVs were present in lysosomal fractions of HBMEC and Caco-2 cells after 8 h, 16 h and 24 h of incubation, but were not found in mitochondrial fractions at any of these time points (Figure 8E, 8F, 8I, 8J). In contrast to OMVs and in accordance with the microscopic analysis (Figure 7C, Figure S5B, S5E), strong EHEC-Hly signals were detected in lysosomal fractions after 8 h, but they significantly decreased after 16 h and were undetectable after 24 h of incubation (Figure 8G, 8H, 8K, 8L). In parallel with its time-dependent disappearance from lysosomes, EHEC-Hly appeared in mitochondria, which contained no EHEC-Hly after 8 h, but clear signals were detected after 16 h and they further significantly increased until 24 h (Figure 8G, 8H, 8K, 8L). No signals with anti-OmpA and anti-EHEC-Hly antibodies were detected in lysosomal and mitochondrial fractions of control, OMV-untreated HBMEC and Caco-2 cells (Figure 6M). The Western blot analyses of purified lysosomal and mitochondrial fractions thus confirmed the CLSM-based results by demonstrating that both OMVs and EHEC-Hly are trafficked to lysosomes, from which EHEC-Hly is subsequently released and translocates to mitochondria. OMVs remain in lysosomes where they are apparently degraded during time as indicated by decreasing OmpA signals in both HBMEC and Caco-2 cells after 16 h and 24 h of incubation (Figure 8I, 8J).

### Bafilomycin A1 inhibits translocation of EHEC-Hly from endo-lysosomal compartments to mitochondria

**Figure 9:**
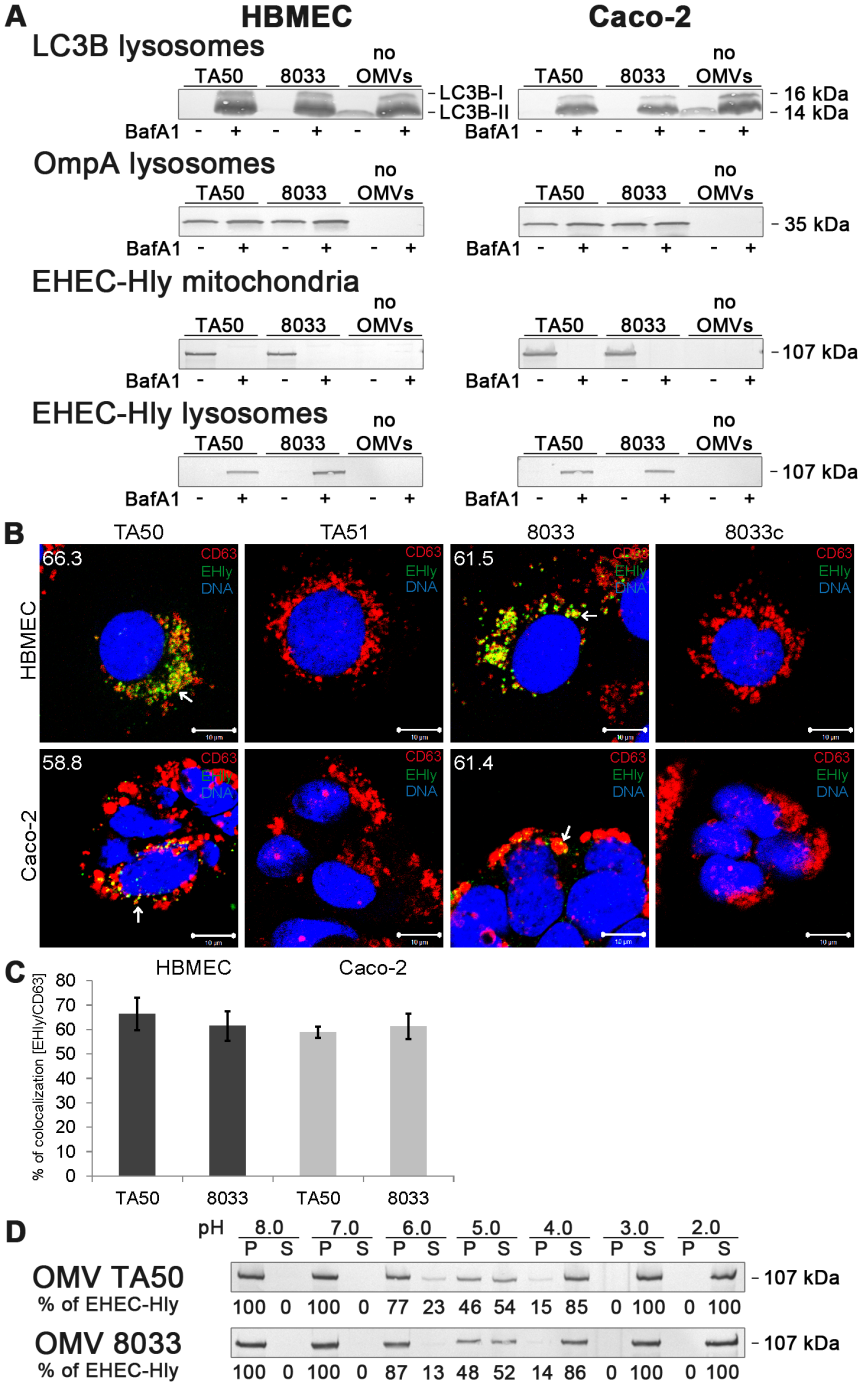
Bafilomycin A1 inhibits translocation of EHEC-Hly from lysosomes to mitochondria. (**A**) HBMEC and Caco-2 cells either pretreated with bafilomycin A1 (BafA1+) (100 nM, 1 h) or BafA1-untreated (BafA1-) were incubated with TA50 or 8033 OMVs or without OMVs for 24 h. Lysosomal and mitochondrial fractions were isolated and analyzed for OMVs and EHEC-Hly using immunoblot with anti-OmpA and anti-EHEC-Hly antibody, respectively. Efficiency of BafA1 treatment was verified using immunoblot with anti-LC3B antibody which detects an increased amount of processed LC3B-II in the presence of BafA1. The sizes of immunoreactive bands are indicated along the right side of Caco-2 cell blots. (**B**) BafA1-pretreated HBMEC and Caco-2 cells were incubated with TA50 or 8033 OMVs (or with control EHEC-Hly-free TA51 or 8033c OMVs) for 24 h and analysed using CLSM. Lysosomes were stained with anti-CD63 antibody and Cy3-conjugated goat anti-mouse IgG (red), EHEC-Hly (EHly) with anti-EHEC-Hly antibody and Alexa Fluor 488-conjugated IgG (green), and nuclei with DRAQ5 (blue). Pictures were taken and processed as described in the legend to Figure 4. Colocalized red and green signals appear in yellow (examples indicated by arrows). The percentages of colocalizations of EHEC-Hly with CD63-positive compartments were calculated using the BioImageXD6 colocalization tool and are shown (averages from at least five different samples) by white numbers in images in panel B and graphically in panel (**C**). Scale bars are 10 µM. (**D**) TA50 and 8033 OMVs were treated (1 h, 37°C) with TRIS-HCl buffer with pH ranging from 8.0 to 2.0; samples were ultracentrifuged and the pellets (P) (containing OMV-associated EHEC-Hly) and supernatants (S) (containing EHEC-Hly that had separated from OMVs) were analyzed for EHEC-Hly using immunoblotting. EHEC-Hly signals in P and S fractions were quantified densitometrically and the percentage of EHEC-Hly present in the P and S fraction at each particular pH was calculated from the total EHEC-Hly signal.

To gain insight into a mechanism involved in translocation of EHEC-Hly from endo-lysosomal compartments to mitochondria, we used bafilomycin A1 (BafA1), a specific inhibitor of vacuolar-type H^+^-ATPase that inhibits acidification of lysosomes by inhibiting the proton pump in their membrane [27]. BafA1-pretreated and control, untreated cells were incubated with TA50 or 8033 OMVs for 24 h and presence of OMVs and EHEC-Hly in lysosomes and mitochondria was determined using Western blot analyses of isolated lysosomal and mitochondrial fractions and CLSM. The efficiency of the BafA1 treatment was verified using immunoblotting with anti-light chain 3B (LC3B) antibody, which detects an increased amount of processed LC3B-II in the presence of BafA1 [28] (Figure 9A). As shown in Figure 9, TA50 and 8033 OMVs were found in lysosomal fractions of HBMEC and Caco-2 cells regardless of absence or presence of BafA1 (Figure 9A, panels OmpA lysosomes), indicating that BafA1 does not influence trafficking of EHEC-Hly-containing OMVs to lysosomes. In contrast, EHEC-Hly, which was after 24 h already present in mitochondria of BafA1-untreated cells (Figure 9A), was found in lysosomal fractions but not in mitochondrial fractions of BafA1-treated cells at this time point (Figure 9A) indicating that the toxin was trapped in lysosomes that failed to acidify. This was supported using CLSM of BafA1-treated HBMEC and Caco-2 cells where EHEC-Hly strongly colocalized with CD63 positive endo-lysosomal compartments after 24 h of incubation (Figure 9B, 9C), in contrast to its absence from lysosomes and presence in mitochondria of BafA1-untreated cells at this time point (Figure 7C, panel 24 h). These experiments showed that endosomal acidification is necessary for EHEC-Hly translocation from endo-lysosomal compartments to mitochondria.

Because EHEC-Hly first needs to be separated from OMVs for translocation to mitochondria, we further tested if EHEC-Hly separation from OMVs is pH-dependent. To this end, we exposed TA50 and 8033 OMVs to a pH range from 8.0 to 2.0 for 1 h, ultracentrifuged the samples and analyzed the presence of EHEC-Hly in pellets (OMV-associated EHEC-Hly) and in supernatants (separated EHEC-Hly) using immunoblot (Figure 9D). In both OMV preparations a pH-dependent dissociation of EHEC-Hly from OMVs occurred. The dissociation started at pH 6.0, reached >50% at pH 5.0 and was almost complete at pH 4.0 (Figure 9D). Because the pH range where EHEC-Hly dissociates from OMVs resembles the acidic pH of lysosomes (∼5.0) [27], this experiment thus indicated that trapping of EHEC-Hly in lysosomes of BafA1-treated cells and the failure of the toxin's translocation to mitochondria is likely to be due to the inability of EHEC-Hly to separate from OMVs in lysosomes that fail to acidify after BafA1 treatment.

### Releasing of EHEC-Hly from lysosomes leads to a transient loss of lysosomal function

**Figure 10:**
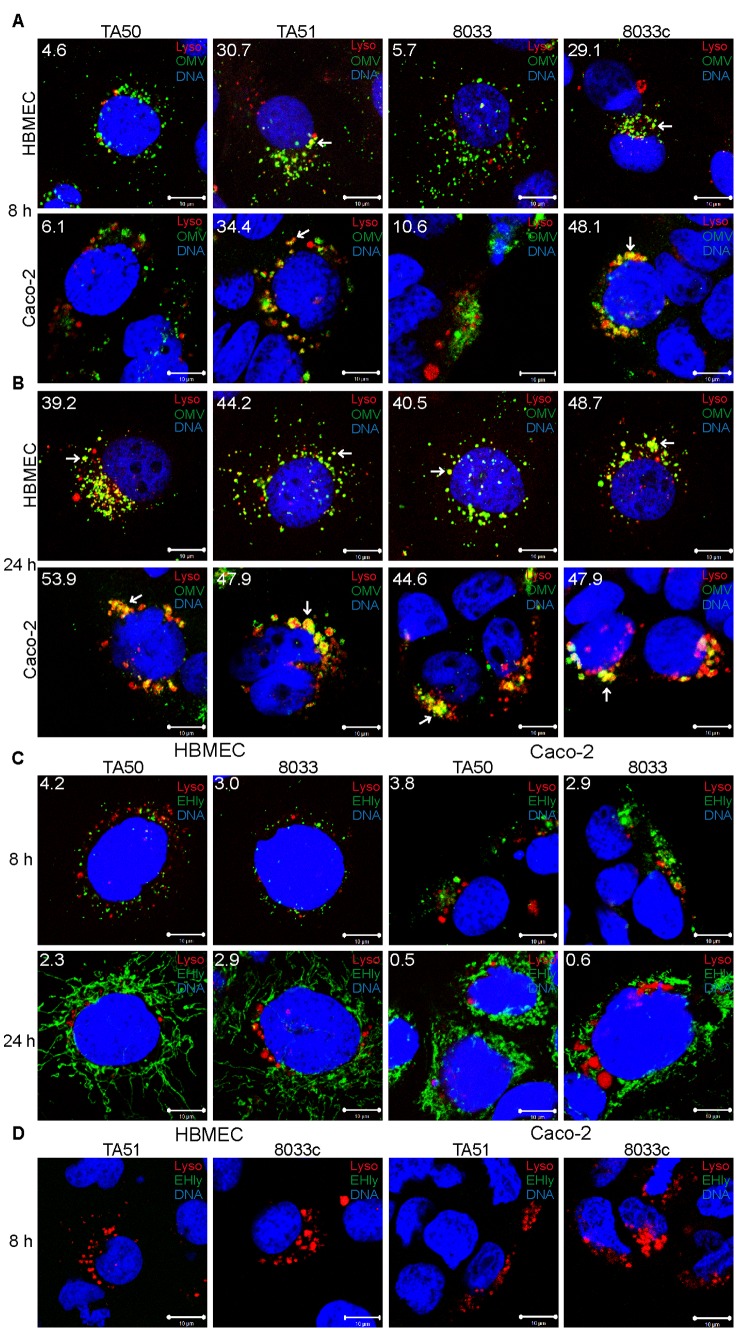
Releasing of EHEC-Hly from lysosomes leads to a transient loss of lysosomal function. (**A, B**) HBMEC and Caco-2 cells were incubated with EHEC-Hly-containing (TA50 or 8033) or EHEC-Hly-free (TA51 or 8033c) OMVs for 8 h (**A**) and 24 h (**B**). OMVs were stained with rabbit anti-*E. coli* LPS antibody and Alexa Fluor 488-conjugated goat anti-rabbit IgG (green), lysosomes with Lysotracker Red DND-99 (red) and nuclei with DRAQ5 (blue). (**C, D**) HBMEC and Caco-2 cells were incubated with TA50 or 8033 OMVs (**C**) or with TA51 or 8033c OMVs (**D**) for 8 h and 24 h. EHEC-Hly (EHly) was stained with rabbit anti-EHEC-Hly antibody and Alexa Fluor 488-conjugated goat anti-rabbit IgG (green), lysosomes with Lysotracker Red DND-99 (red), and nuclei with DRAQ5 (blue). Pictures were taken and processed as described in the legend to Figure 4. Colocalized red and green signals appear in yellow (examples in panels A and B are depicted by arrows). White numbers indicate the percentages of OMVs or EHEC-Hly colocalized with Lysotracker Red DND-99-positive lysosomes (averages from at least five different samples) calculated using the BioImageXD6 colocalization tool. Scale bars are 10 µm. The pictures shown in panel D (8 h of incubation) are also representative of 24 h (no EHEC-Hly was detected in cells treated with EHEC-Hly-free OMVs at any of these time points).

Following its separation from OMVs in acidified lysosomes, EHEC-Hly has to escape from lysosomes in order to reach mitochondria. Because EHEC-Hly is a pore-forming toxin we hypothesized that it might be released from lysosomes by damaging the lysosomal membrane. To test this hypothesis, we incubated HBMEC and Caco-2 cells with EHEC-Hly-containing OMVs TA50 or 8033 or with EHEC-Hly-free OMVs TA51 or 8033c (used as controls) for 8 h, 16 h, and 24 h, and analyzed the colocalization of OMVs and EHEC-Hly with functional lysosomes using lysotracker. Since lysotracker detects only lysosomes that retain their acidic (physiological) pH, the detection of lysosomes by lysotracker would be diminished in the case of EHEC-Hly-mediated lysosomal pore formation and the resulting increase in lysosomal pH. Indeed, after 8 h of incubation, when EHEC-Hly is detected together with OMVs in CD63-positive endo-lysosomal compartments (Figure 7A, 7C, panels 8 h) and in isolated lysosomes (Figure 8E-8H, 8 h), we observed only a low degree of colocalization of TA50 and 8033 OMVs with functional/acidified lysosomes stained by lysotracker in both HBMEC (4.6% and 5.7%, respectively) and Caco-2 cells (6.1% and 10.6%, respectively) (Figure 10A). In contrast, EHEC-Hly-free TA51 and 8033c OMVs demonstrated significantly higher colocalization with lysotracker-stained lysosomes in both HBMEC (30.7% and 29.1%, respectively) and Caco-2 cells (34.4% and 48.1%, respectively) (Figure 10A, Figure S6C). These observations suggested an EHEC-Hly-mediated effect on the acidification of lysosomes. Notably, the colocalization rates of TA50 and 8033 OMVs with functional lysosomes increased after 16 h and 24 h of incubation, when they were nearly identical to those of TA51 and 8033c OMVs (Figure 10B, Figure S6A, S6C). This correlated with gradual disappearance of EHEC-Hly from CD63-positive compartments (Figure 7C, panel 24 h, Figure S5B, S5E) and from isolated lysosomes (Figure 8G, 8H, 8K, 8L, 16 h and 24 h) at these time points. This suggested that the ability of lysosomes to acidify was restored after EHEC-Hly had been released. The involvement of EHEC-Hly in the impaired acidification of lysosomes caused by TA50 and 8033 OMVs was directly supported by the observation that EHEC-Hly did not colocalize with lysotracker-stained lysosomes after 8 h of incubation (Figure 10C, panel 8 h), although it was present in lysosomes stained with anti-CD63 antibody (Figure 7C, panel 8 h, Figure S5E) and in isolated lysosomal fractions (Figure 8G, 8H, 8K, 8L, 8h) at this time. Compared to this, the lack of colocalization of EHEC-Hly with lysotracker-stained lysosomes at later time points (16 h and 24 h; Figure S6B and Figure 10C, panel 24 h, respectively) resulted from a significant decrease of the toxin amount (16 h) and its absence from lysosomes (24 h) (Figure S5B, S5E, Figure 7C, panel 24 h; Figure 8G, 8H, 8K, 8L), which was due to EHEC-Hly translocation to mitochondria (Figure S5B, Figure 7C, panel 24 h; Figure 8G, 8H, 8K, 8L). Taken together, these experiments suggest that EHEC-Hly is released from lysosomes via its pore-forming activity which might perforate the lysosomal membrane, thereby enabling the toxin to escape and subsequently translocate to mitochondria.

### Translocation of EHEC-Hly into mitochondria permeabilizes the mitochondrial membranes in HBMEC and Caco-2 cells

**Figure 11:**
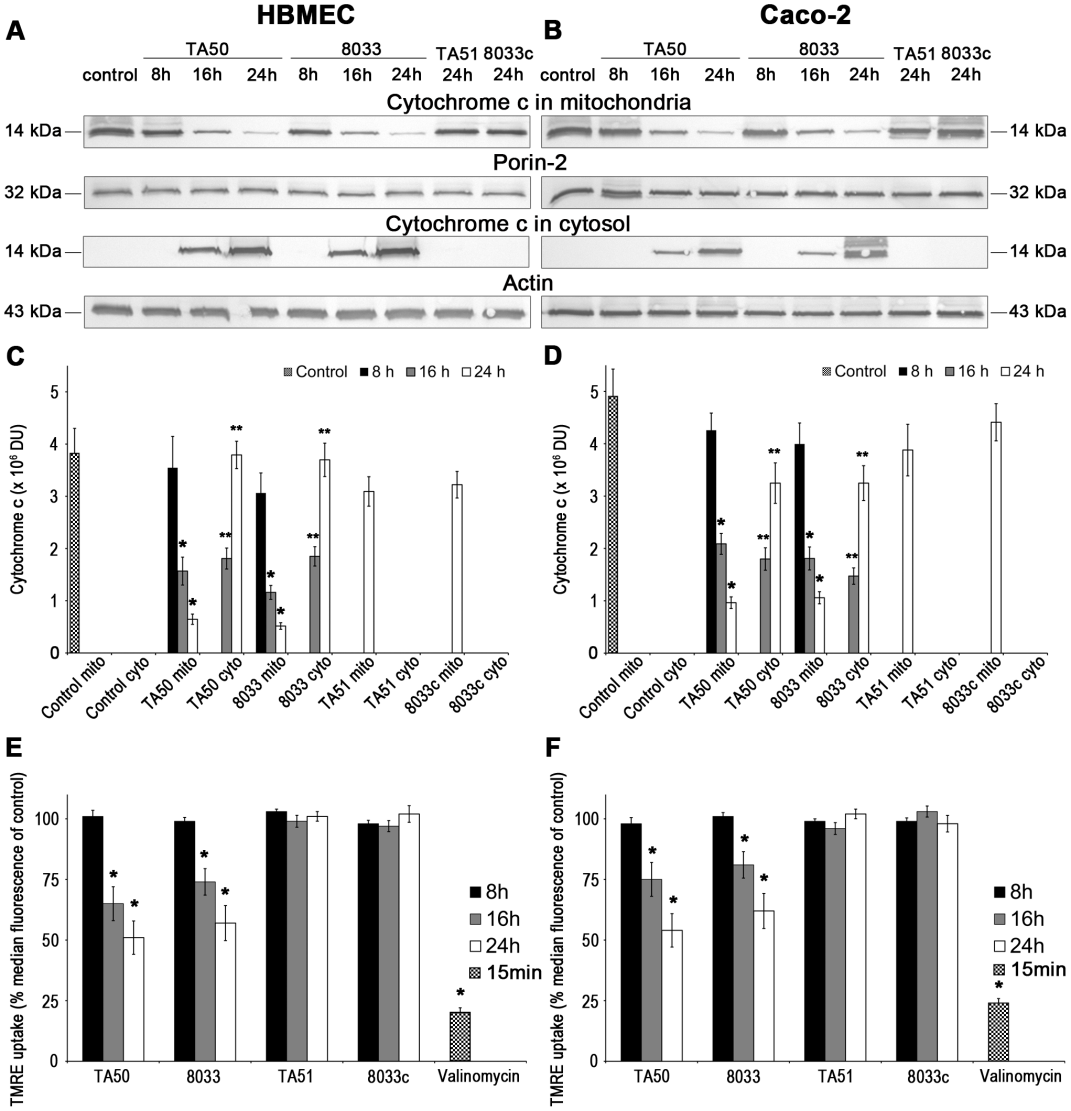
Translocation of EHEC-Hly to mitochondria results in cytosolic cytochrome c release and ΔΨ_m_ decrease. (**A, B**) HBMEC (**A**) and Caco-2 cells (**B**) were incubated for the times indicated with EHEC-Hly-containing OMVs TA50 or 8033 (20 µg of OMV protein containing 100 ng of EHEC-Hly) or with corresponding protein amounts of EHEC-Hly-negative OMVs TA51 or 8033c or left untreated (control). Mitochondrial and cytosolic fractions were isolated and immunoblotted with anti-cytochrome c antibody. After stripping, membranes were reprobed with antibody against porin-2 (mitochondrial marker) or actin (cytosolic marker). The sizes of immunoreactive bands are indicated. (**C, D**) Intensities of the cytochrome c signals in mitochondrial (mito) and cytosolic (cyto) fractions shown in panels A and B were quantified in HBMEC (**C**) and Caco-2 cells (**D**) using densitometry, expressed in arbitrary densitometric units (DU) and are presented as means ± standard deviations from three independent experiments. * Significantly decreased (*p*<0.05) compared to mitochondrial cytochrome c in control cells; ** significantly increased (*p*<0.05) compared to cytosolic cytochrome c in control cells (unpaired Student's *t* test). Mitochondrial cytochrome c signals in control cells (control mito) were almost identical after 8 h, 16 h and 24 h and are therefore shown as means ± standard deviations from all times. No cytochrome c was detected at any time in cytosol of control cells (control cyto). (**E, F**) HBMEC (**E**) and Caco-2 cells (**F**) were incubated with EHEC-Hly-containing or EHEC-Hly-free OMVs as indicated in A and B and ΔΨ_m_ was determined by uptake of TMRE using flow cytometry. Median fluorescence of OMV-treated cells was expressed as the percentage of the median fluorescence of control untreated cells (defined as 100%). The values represent means ± standard deviations from three independent experiments. * Significantly decreased (*p*<0.05) compared to control cells (unpaired Student's *t* test). Valinomycin (100 nM, 15 min) was a positive control.

Since mitochondria are central components in intrinsic apoptosis [29], we investigated if translocation of EHEC-Hly into mitochondria triggers this pathway. The key event in the intrinsic apoptotic pathway is the permeabilization of the mitochondrial membranes; the outer membrane permeabilization leads to the release of pro-apoptotic proteins (such as cytochrome c) from the mitochondrial intermembrane space to the cytosol, and permeabilization of the inner membrane dissipates the mitochondrial transmembrane potential (ΔΨ_m_) [29]. Incubation of HBMEC and Caco-2 cells with TA50 or 8033 OMVs for 8 h, 16 h and 24 h resulted in a steady decrease of cytochrome c in the mitochondrial fractions and a steady increase of cytochrome c in the cytosolic fractions (Figure 11A-11D). The kinetics of the cytochrome c release from mitochondria to the cytosol correlated with the kinetics of EHEC-Hly separation from OMVs and its translocation to mitochondria (Figure 5A, panels 8 h, 16 h, 24 h). Moreover, TA50 and 8033 OMVs caused a time dependent decrease in uptake of tetramethylrhodamine ethyl ester (TMRE), a mitochondrial potential-sensitive dye, by HBMEC and Caco-2 cells (Figure 11E, 11F). After 24 h, the TMRE uptake dropped to 52% (TA50 OMVs) and 57% (8033 OMVs) of control in HBMEC, and to 54% (TA50 OMVs) and 62% (8033 OMVs) of control in Caco-2 cells (Figure 11E, 11F). No change in TMRE uptake and no cytochrome c release from mitochondria to the cytosol was elicited by EHEC-Hly-free TA51 and 8033c OMVs (Figure 11A-11F). Thus, translocation of EHEC-Hly to mitochondria results in release of cytochrome c and a decrease of ΔΨ_m_ supporting the toxin-mediated permeabilization of the outer and the inner mitochondrial membrane.

### EHEC-Hly induces activation of caspase-9 and caspase-3, but not of caspase-8

**Figure 12:**
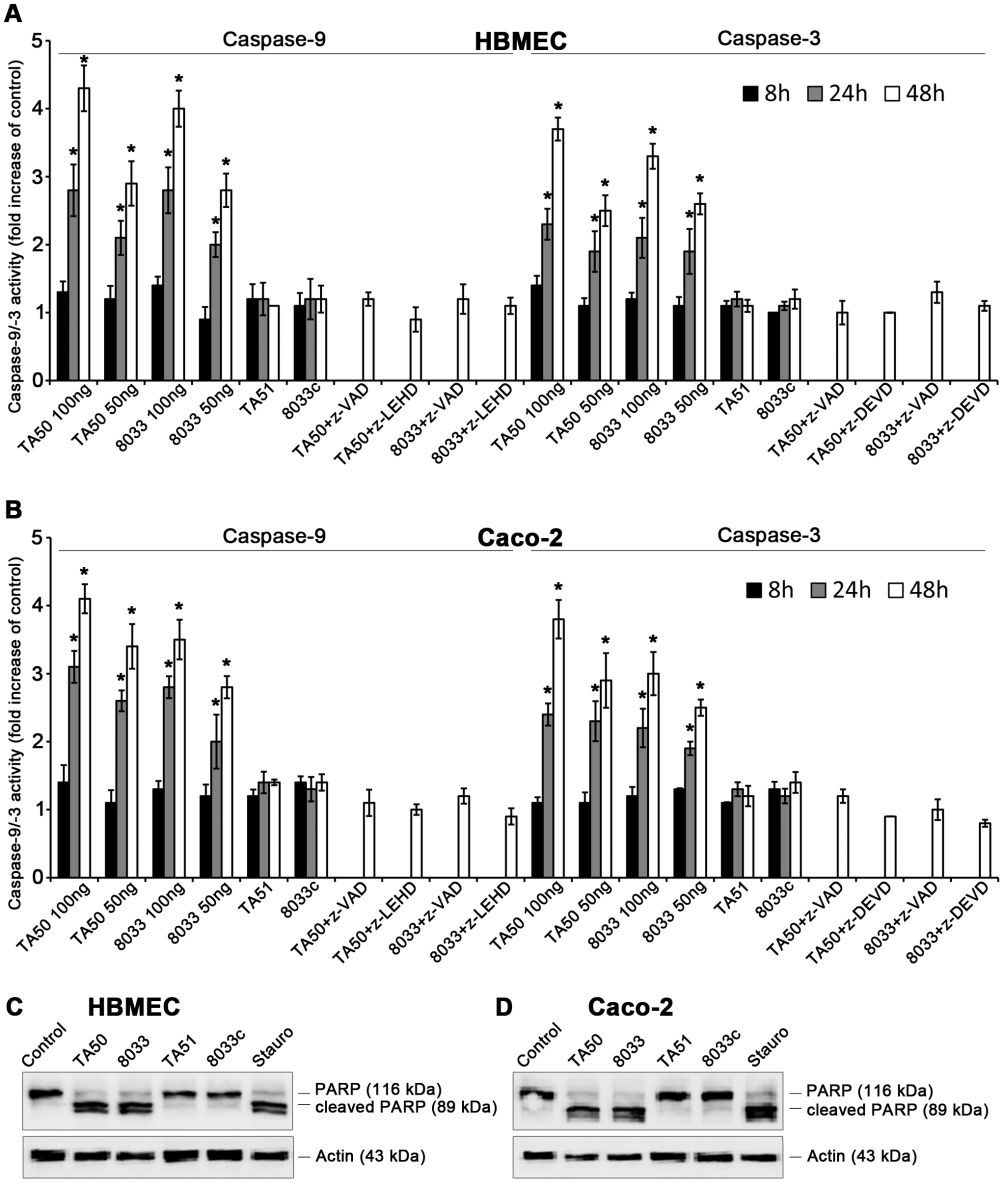
EHEC-Hly induces activation of caspase-9 and caspase-3 and PARP cleavage. (**A, B**) HBMEC (**A**) and Caco-2 cells (**B**) were incubated with different doses of TA50 or 8033 OMVs (20 µg or 10 µg of OMV protein containing 100 ng or 50 ng of EHEC-Hly, respectively) or with 20 µg of EHEC-Hly-negative OMVs (TA51 or 8033c) for the times indicated or remained untreated. Cells were lysed, the lysates were incubated with colorimetric substrates of caspase-9 (Ac-LEHD-pNA) or caspase-3 (Ac-DEVD-pNA) and the color intensity, which is proportional to the level of caspase enzymatic activity, was measured spectrophotometrically. The activity of each caspase in OMV-treated cells was expressed as a fold-increase of that in untreated cells (defined as 1). Specific inhibitors of caspase-9 (z-LEHD-fmk), caspase-3 (z-DEVD-fmk) or pan-caspase inhibitor z-VAD-fmk were added to cells 30 min before treatment with OMVs containing 100 ng of EHEC-Hly. Data are shown as means ± standard deviations from three independent experiments. * Significantly increased (*p*<0.05) compared to control cells (unpaired Student's *t* test). (**C, D**) HBMEC (**C**) and Caco-2 cells (**D**) were incubated with TA50 or 8033 OMVs (100 ng of EHEC-Hly) or with TA51 or 8033c OMVs (20 µg of OMV protein) for 48 h or remained untreated (negative control). Cells treated with 1 µM staurosporin (Stauro) for 3 h served as a positive control. Presence of uncleaved PARP (116 kDa) and the PARP cleavage product (89 kDa) in cell lysates was determined using an immunoblot with anti-PARP antibody; anti-actin antibody was used as a loading control. The results are representative of two independent experiments.

Upon its release from mitochondria to the cytosol, cytochrome c binds together with the apoptosis-activating factor-1 and ATP/dATP to pro-caspase-9, leading to its cleavage and activation [29], [30]. Active caspase-9, the initiator caspase of the intrinsic apoptotic pathway, cleaves and activates effector caspases including caspase-3 [29], [30]. EHEC-Hly-containing OMVs TA50 and 8033 caused time- and dose-dependent activation of caspase-9 and caspase-3 in HBMEC and Caco-2 cells, which peaked after 48 h (Figure 12A, 12B). At this point the activity of caspase-9 was up to 4.3-fold increased in HBMEC and up to 4.1-fold increased in Caco-2 cells, and the activity of caspase-3 was up to 3.7-fold increased in HBMEC and up to 3.8-fold increased in Caco-2 cells compared to those in control, OMV-untreated cells (Figure 12A, 12B). In contrast, the activity of caspase-8, the initiator caspase of the extrinsic apoptotic pathway [31], in cells treated with TA50 or 8033 OMVs remained at baseline (<1.2-fold increase) for the duration of the experiment (Figure S7). Activation of caspase-9 and caspase-3 in each cell line was inhibited by pre-treatment with the specific caspase inhibitors (z-LEHD-fmk and z-DEVD-fmk, respectively) and with the pan-caspase inhibitor z-VAD-fmk (Figure 12A, 12B). No activation of caspase-9 or caspase-3 was observed in cells treated with EHEC-Hly-free OMVs TA51 or 8033c (Figure 12A, 12B).

In accordance with activation of caspase-3, the poly-(ADP-ribose) polymerase (PARP), one of the major targets of caspase-3 in mammalian cells [32], was cleaved in HBMEC and Caco-2 cells treated with TA50 or 8033 OMVs (Figure 12C, 12D). EHEC-Hly-free TA51 and 8033c OMVs, which did not activate caspase-3 (Figure 12A, 12B), did not cleave PARP in any cell line (Figure 12C, 12D). Hence, translocation of EHEC-Hly to mitochondria and the resulting cytochrome c release into the cytosol activates caspase-9, but not caspase-8, and triggers the intrinsic apoptotic pathway.

### EHEC-Hly triggers DNA fragmentation in HBMEC and Caco-2 cells

**Figure 13:**
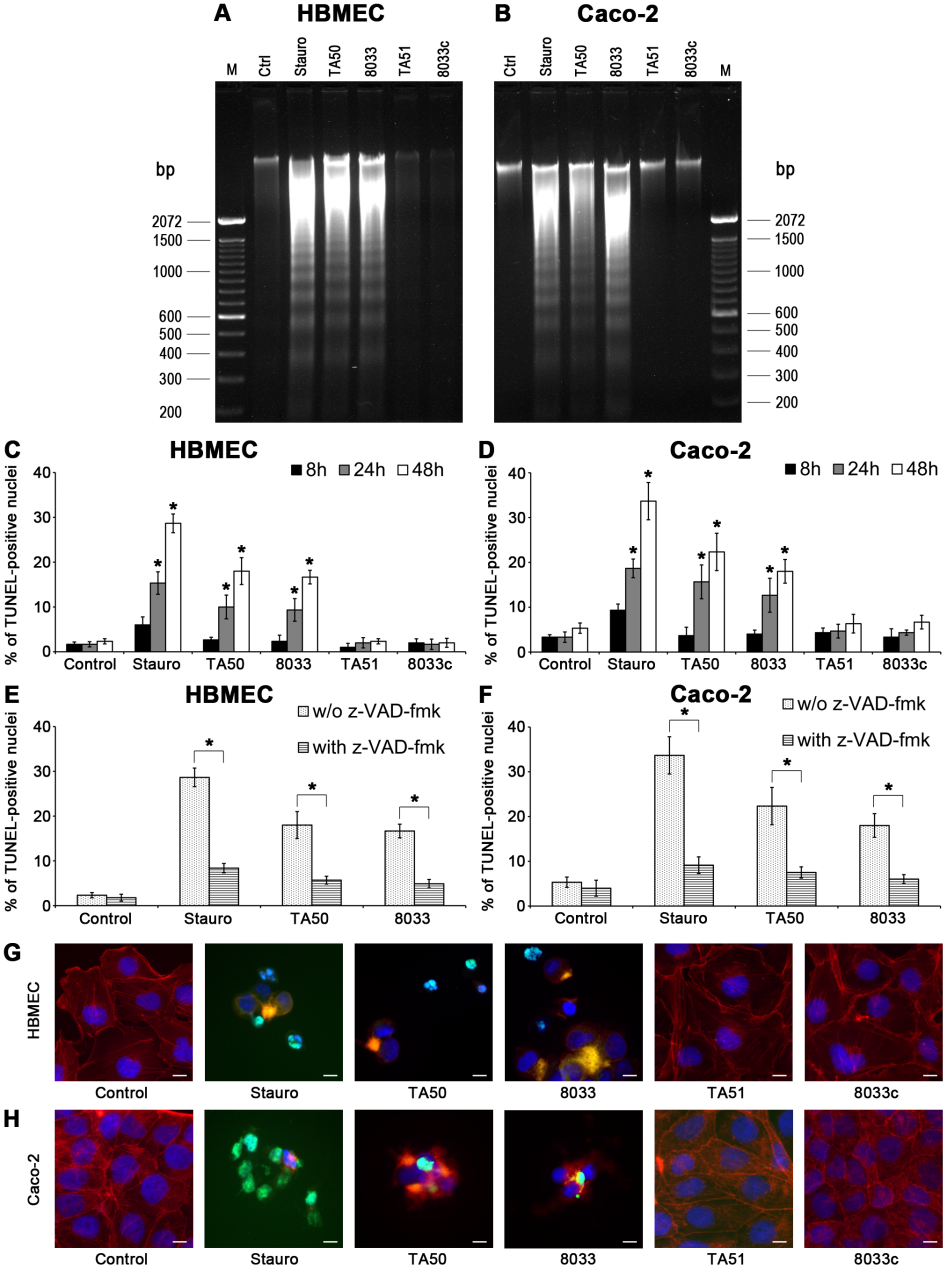
EHEC-Hly-containing OMVs cause DNA laddering and formation of TUNEL-positive nuclei. (**A, B**) HBMEC (**A**) and Caco-2 cells (**B**) were incubated with EHEC-Hly-containing OMVs (TA50 or 8033; 100 ng of EHEC-Hly) or EHEC-Hly-free OMVs (TA51 or 8033c; 20 µg of OMV protein) for 48 h. Cellular DNA was separated on agarose gel and visualized after staining with Midori Green Advance. M, molecular size marker (100 bp DNA ladder). Untreated cells (Ctrl) were a negative control and cells treated with 1 µM staurosporin (Stauro) a positive control. (**C, D**) HBMEC (**C**) and Caco-2 cell (**D**) were incubated for the times indicated with TA50, 8033, TA51 or 8033c OMVs in the amounts shown above or with 1 µM staurosporin or remained untreated (control). After TUNEL reagent staining, the proportions of TUNEL-positive nuclei were determined by fluorescence microscopy and expressed as the percentage of total number of cells examined. Data are means ± standard deviations from three independent experiments. * Significantly increased (*p*<0.05) compared to control cells (unpaired Student's *t* test). (**E, F**) HBMEC (**E**) and Caco-2 cells (**F**), either without or after pretreatment with pan-caspase inhibitior z-VAD-fmk were incubated for 48 h with TA50 or 8033 OMVs or with 1 µM staurosporin and the percentages of TUNEL-positive nuclei were determined as described above. Data are means ± standard deviations from three independent experiments. * Significantly decreased (*p*<0.05) compared to non-pretreated cells (unpaired Student's *t* test). (**G, H**). Photomicrographs of TUNEL reagent-stained HBMEC (**G**) and Caco-2 cells (**H**) incubated for 48 h with the probes indicated or remained untreated (control). TUNEL-positive nuclei stain green, other nuclei blue (DAPI) and actin red (phalloidin-TRITC). Bars are 10 µm.

We further analyzed HBMEC and Caco-2 cells treated with TA50 or 8033 OMVs for DNA fragmentation, the signature of the apoptotic phenotype [29], [31], [33]. Each of the OMVs elicited a characteristic DNA ladder pattern resulting from internucleosomal DNA cleavage during apoptosis [33] in each cell line (Figure 13A, 13B). Similar DNA patterns were observed in cells treated with the apoptosis-inducing reagent staurosporin. In contrast, no DNA ladder appeared in cells treated with EHEC-Hly-free OMVs TA51 or 8033c (Figure 13A, 13B), indicating that EHEC-Hly triggers the mechanism(s) leading to DNA fragmentation.

DNA fragmentation in individual cells was visualized using TUNEL (terminal deoxynucleotidyl transferase [TdT]-mediated dUTP nick end labeling) assay [34]. HBMEC and Caco-2 cells were exposed to TA50 or 8033 OMVs for 8 h to 48 h and the percentage of TUNEL-positive nuclei was determined microscopically. In each cell line, the percentage of TUNEL-positive nuclei increased over time and peaked after 48 h (Figure 13C, 13D). Pretreatment with the pan-caspase inhibitor zVAD-fmk significantly reduced the percentage of TUNEL-positive nuclei (Figure 13E, 13F). In cells treated with EHEC-Hly-free TA51 or 8033c OMVs the percentage of TUNEL-positive nuclei remained at the basal level of untreated cells during the whole experiment (Figure 13C, 13D). A detailed microscopic analysis of HBMEC and Caco-2 cells treated with TA50 or 8033 OMVs (Figure 13G, 13H) demonstrated that in addition to TUNEL-positive nuclei, the cells displayed other typical morphological signs of apoptosis including fragmented and/or condensed nuclei and cytoplasmic reduction or loss. Thus, activation of caspase-9 induced by EHEC-Hly leads to apoptotic death of HBMEC and Caco-2 cells.

## Discussion

**Figure 14:**
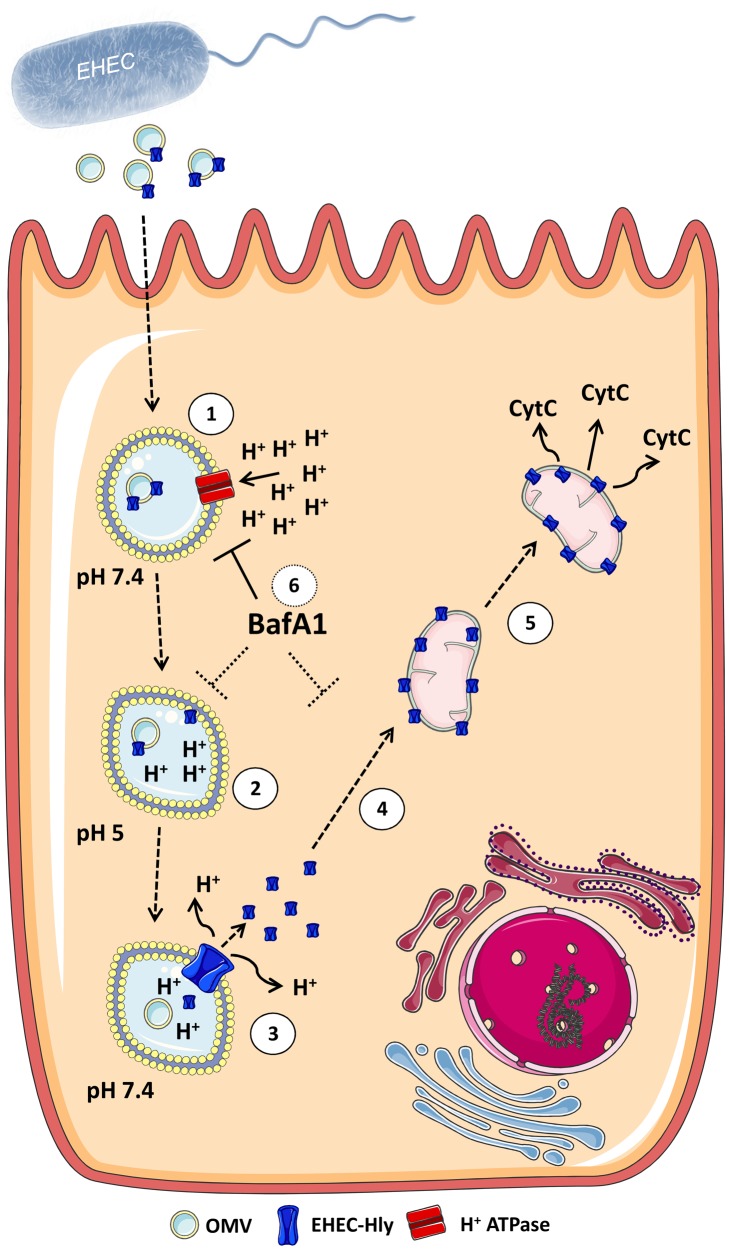
Model of intracellular trafficking and action of OMV-associated EHEC-Hly. 1. After its secretion by EHEC bacteria and association with OMVs, the OMV-associated EHEC-Hly is endocytosed by dynamin-dependent endocytosis and enters the endosomal compartments of target cells. 2. During endosome acidification via the H^+^-ATPase the neutral pH 7.4 of endosomes drops to pH 5.0, which induces separation of EHEC-Hly from OMVs. 3. The separated toxin plausibly interacts with the endosomal/lysosomal membrane and, as a pore-forming toxin, it damages lysosomal membrane by its pore-forming activity in order to release from lysosomes. As a consequence of the membrane damage, the proton gradient of lysosomes is disrupted leading to lysosomal pH increase. 4. EHEC-Hly released from lysosomes translocates by an unknown mechanism to mitochondria. 5. This results in cytochrome c release to the cytosol, which leads to activation of caspase-9 and caspase-3 and apoptotic cell death. 6. Presence of the proton ATPase inhibitor BafA1 inhibits endosomal acidification and thus prevents the toxin to be separated from OMVs. As a consequence, EHEC-Hly is trapped in endosomes/lysosomes and cannot translocate into mitochondria. The figure was produced using Servier Medical Art.

OMVs ubiquitously shed by Gram-negative bacteria are emerging as a new, highly sophisticated mechanism for secretion and a simultaneous, coordinated and direct delivery of bacterial virulence factors into host cells [19], [20], [35]–[38]. OMVs, which contain bacterial toxins, adhesins, invasins, and immunomodulatory compounds are considered as bacterial weapons that attack the host tissues and assist bacterial pathogens to establish their colonization niches, impair host cellular functions, and modulate host defense [reviewed in 39–41]. We now demonstrate that OMV-associated EHEC-Hly irreversibly injures human microvascular endothelial and intestinal epithelial cells, which are key players in the pathogenesis of EHEC-mediated diseases. Our data support the model (Figure 14) that the toxin is internalized via OMVs by dynamin-dependent endocytosis and trafficked together with its carriers to endo-lysosomal compartments (Figure 14, Step 1). During endosomal acidification EHEC-Hly is separated from OMVs (Step 2), escapes from the lysosomes, most probably via its pore-forming activity towards the lysosomal membrane (Step 3), and targets mitochondria (Step 4). The presence of EHEC-Hly in mitochondria leads to a reduction of the mitochondrial transmembrane potential and release of cytochrome c to the cytosol (Step 5). Subsequent activation of caspase-9 triggers the apoptotic cell death.

Our study provides the first evidence that EHEC-Hly, when it is delivered into the host cells via OMVs, targets mitochondria and acts pro-apoptotically. To our knowledge, EHEC-Hly is the first virulence factor in general and the first RTX toxin in particular, which employs OMVs for its intracellular delivery in order to subsequently hijack mitochondria. Although several other bacterial proteins target mitochondria and cause their dysfunction [42]–[51], these molecules enter cells by other, OMV-independent mechanisms including receptor-mediated intracellular delivery or cytosolic injection via the type III secretion system [43], [49], [52]–[55]. Hence, the OMV-mediated delivery represents a novel mechanism for a bacterial toxin to enter the host cells in order to subvert mitochondrial function thereby causing cell death.

In contrast to the previously reported lytic effect of free EHEC-Hly on microvascular endothelial cells [6], the OMV-associated EHEC-Hly does not lyse either HBMEC or Caco-2 cells, even though it causes hemolysis. One reason could be the difference between the dose of free EHEC-Hly which is required for efficient endothelial cell lysis (∼40 µg/ml) [6] and the amount of EHEC-Hly associated with OMVs (∼3 µg/ml). This dose-dependent mechanism of cytotoxicity of EHEC-Hly resembles that of other RTX toxins, which in high doses lyse the target cells via their pore-forming activity, whereas in low, sublytic doses they cause a variety of other biological effects including apoptosis [45], [54], [56]–[62]. Although the mechanism underlying this phenomenon is unknown, differential extents of perturbation of mitochondrial function, which is the key factor determining the route of cell death (i.e. necrosis versus apoptosis), after exposure to the high and to the low toxin doses could play a role [54], [63]. We hypothesize that OMV-associated EHEC-Hly, rather than free EHEC-Hly, may be the pathophysiologically relevant form of the toxin because: i) free EHEC-Hly rapidly and irreversibly loses biological activity *in vitro* at 37°C [64] making it questionable if the toxin can reach host cells in active form *in vivo* during infection; ii) free EHEC-Hly has a high affinity to OMVs leading to rapid OMV association (Figure 1B–1E) [16], which increases its stability and prolongs its biological activity [16]; iii) the OMV-associated EHEC-Hly injures the cells involved in the pathogenesis of EHEC-mediated diseases.

OMV-associated EHEC-Hly is internalized via OMVs and the toxin itself is not required for OMV internalization involving dynamin-dependent and clathrin-mediated endocytosis (Figure 4A, 4B, 4D). This is similar to OMVs from *Aggregatibacter actinomycetemcomitans*, which also associate with target cells regardless of the presence of their cargo toxins [19], [65]. In contrast, the cell binding and internalization of OMVs containing heat labile enterotoxin (LT) of enterotoxigenic *E. coli* [20] or cholera toxin [37] occur via interactions of the OMV-associated toxins with their specific cell surface receptor (GM1 in both cases) and are therefore toxin-dependent [20], [37]. Interestingly, the dispensability of EHEC-Hly for adherence and internalization of EHEC-Hly-harboring OMVs by HBMEC and Caco-2 cells is in strong contrast with its mandatory requirement for interaction of EHEC-Hly-containing OMVs with erythrocytes, in which EHEC-Hly functions both as a cell-binding protein and a hemolysin [16]. This indicates that EHEC-Hly-containing OMVs use different mechanisms in their interactions with erythrocytes and nucleated cells and that other OMV components than EHEC-Hly are involved in association of such OMVs with HBMEC and Caco-2 cells. Potential candidates for such molecules are various OMV membrane proteins and/or LPS [21], [66], [67]. Mechanism(s) by which EHEC-Hly might contribute to OMV cellular binding/internalization as suggested by our flow cytometric analyses (Figure 3A, 3B) need(s) to be determined in future studies. A supportive role in uptake of OMVs from *Helicobacter pylori* by gastric cells has also been proposed for vacuolating toxin A (VacA) [21].

Interestingly, we observed that endosomal acidification is an essential mechanism for EHEC-Hly to separate from OMVs and escape from lysosomes, which can be blocked by BafA1 treatment (Figure 14, Step 6). As a paradox, we never observed EHEC-Hly colocalized with acidified lysosomes stained by lysotracker (Figure 10C, Figure S6B). However, we believe that EHEC-Hly as a pore-forming toxin, once separated from OMVs by a pH drop, disrupts the endo-lysosomal membrane, releasing the toxin and protons from lysosomes (Figure 14, Step 3). As a consequence of membrane damage, the proton gradient of lysosomes is disrupted, which leads to lysosomal pH increase and thus excludes detection of functional lysosomes by lysotracker. This hypothesis is supported by the fact, that after EHEC-Hly had been released from lysosomes, their function was restored (Figure 10B).

In contrast to its OMV-mediated intracellular delivery, translocation of EHEC-Hly from lysosomes to mitochondria does not require OMVs and is likely to be related to specific characteristics of the toxin itself. A putative mitochondrial targeting signal (MTS) was recently identified in the N-terminal region of EHEC-Hly sequence [50], though the ability of the toxin to target mitochondria was not investigated. MTSs are also present in N-terminal regions of secreted proteins EspF [47], [68] and Map [48], [49] of enteropathogenic *E. coli* (EPEC), leukotoxin of *Mannheimia hemolytica* [50], and ApxII exotoxin of *Actinobacillus pleuropneumoniae* [50] and direct these proteins to mitochondria [47], [48], [50] likely exploiting the endogenous import machinery for nuclear-encoded mitochondrial proteins [42], [43], [69]. The presence of MTS in the EHEC-Hly molecule thus provides a mechanistical basis for our evidence of the toxin's mitochondria-targeting activity.

The ability of EHEC-Hly to induce translocation of cytochrome c from mitochondria to the cytosol and decrease of ΔΨ_m_ suggests that the toxin permeabilizes the outer and the inner mitochondrial membranes. However, the mechanism(s) of interaction of EHEC-Hly with mitochondria is presently unknown. Two major pathways involved in mitochondrial sorting of bacterial virulence factors possessing the MTS have been identified [42], [43]. Proteins that enter the intermembrane space translocate via the translocase of the outer membrane (TOM) complex, whereas proteins that integrate into the inner mitochondrial membrane or enter the mitochondrial matrix interact with the translocase of the inner membrane (TIM) complex [42]. EspF and Map enter mitochondria via the TOM complex and are subsequently sorted to the mitochondrial matrix via the TIM complex [42], [48], [68]. Because the TOM machinery is the most common pathway for mitochondrial delivery of MTS-possessing proteins [42], [69], the presence of MTS in the EHEC-Hly molecule suggests that EHEC-Hly might be imported in mitochondria via the TOM complex. Moreover, the presence of a putative cleavage site in the EHEC-Hly MTS [50], which is also present in the EspP and Map MTSs [47]–[49] and is indicative of proteins' import across the mitochondrial membranes [69] suggests that EHEC-Hly may be transported into the mitochondrial matrix [50]. However, as for many other bacterial virulence factors that target mitochondria [42], the mitochondrial sorting route of EHEC-Hly and the mechanism of the mitochondrial injury are not yet determined.

The intracellular trafficking of OMV-delivered EHEC-Hly and the development of the EHEC-Hly-mediated apoptotic effects are delayed compared to such effects of other mitochondria-targeting virulence factors, which are delivered into the host cells as free proteins via a specific receptor or via the type III secretion system [52], [53]. Whereas EHEC-Hly was first detected in mitochondria after 16 h (Figure 5A), when also cytochrome c translocation and ΔΨ_m_ reduction were observed (Figure 11), leukotoxin of *M. hemolytica* colocalized with mitochondria of BL-3 cells already after 30 min of incubation [46] and cytochrome c cytosolic translocation, ΔΨ_m_ decrease and cell death occurred between 4 h and 6 h [45]. Similarly, sorting of EspF to mitochondria, loss of ΔΨ_m_ and cytosolic translocation of cytochrome c were observed after 1 h of incubation of cells with EspF-producing EPEC strains [47]. The delayed apoptotic effects of OMV-associated EHEC-Hly are in accordance with the delayed hemolysis caused by this toxin form (Figure S1E and [16]), which contrasts with the rapid hemolysis caused by free EHEC-Hly [16]. We hypothesized [16] that the delayed hemolytic effect of OMV-associated EHEC-Hly is due to structural rearrangement of the OMV-associated toxin before it can bind and lyse erythrocytes. Because OMV/EHEC-Hly complexes internalize rapidly (Figure 3A, 3B), the delay in the onset of apoptotic effects may be due to: i) the intracellular trafficking of OMV-associated EHEC-Hly to endo-lysosomes; ii) the endosomal acidification-dependent separation of EHEC-Hly from OMVs and its putative conformational change, which is plausibly required for its ability to injure the lysosomal membrane and the release from lysosomes; and iii) the translocation of the toxin to mitochondria and disruption of the mitochondrial function. The proposed conformational changes may lead to the exposure of the pore-forming region located in RTX toxins in their N-terminal region [54]. This pore-forming region is likely to be masked in OMV-associated EHEC-Hly during contact with cellular membrane resulting in the failure of this toxin form to cause cell lysis.

In conclusion, EHEC-Hly delivered into the host cells via OMVs targets mitochondria and triggers apoptosis of human microvascular endothelial and intestinal epithelial cells, representing a novel mechanism for the toxin's involvement in the EHEC pathogenesis. Mechanisms of EHEC-Hly-mediated mitochondrial injury warrant further investigations.

## Materials and Methods

### Bacterial strains

TA50 is *E. coli* K-12 strain MC1061 transformed with pBlueskript KS II(+) (Stratagene, La Jolla, Ca., USA) harboring the EHEC-*hlyCABD* operon from an HUS-associated *E. coli* O26:H11 [16]. TA51 (*E. coli* K-12 MC1061 transformed with pBlueskript KS II(+) alone) served as a vector control. Strain 8033 is an EHEC O103:H2 isolate from a patient with HUS that lost the Stx-encoding bacteriophage and Stx production [14]. The strain was selected for the study because of its strong enterohemolytic phenotype (Figure 1A) and because the absence of Stx prevents an interference of cellular effects of OMV-associated EHEC-Hly with those of Stx. Strain 8033c (cured) is a spontaneous derivative of strain 8033, which lost the EHEC-Hly-encoding gene and hereby EHEC-Hly production during *in vitro* passaging.

### Isolation of OMVs, protein concentration and EHEC-Hly content

OMVs were isolated as described previously [16] with slight modifications. Briefly, bacterial strains were grown in 500 ml of LB broth (supplemented with 100 µg/ml of ampicillin for strains TA50 and TA51) for 15 h (37°C, 180 rpm). Bacteria were removed by centrifugation (9,800× g, 10 min, 4°C) and supernatants were filtered using Stericup filters (pore size 0.22 µm) (Millipore Corporation, Billerica, MA, USA). OMVs were collected from the filtered supernatants by ultracentrifugation (235,000× g, 2 h, 4°C) in a 45 Ti rotor (Beckman Coulter, Krefeld, Germany) and the OMV pellets were resuspended in 1 ml of 20 mM TRIS-HCl (pH 8.0). Aliquots of OMV preparations were inoculated on blood agar to verify their sterility. Protein concentration in OMV preparations was determined using the Roti-Nanoquant reagent (Carl Roth, Karlsruhe, Germany) and was 604±123 µg/ml, 580±161 µg/ml, 610±149 µg/ml, and 572±108 µg/ml in OMVs TA50, TA51, 8033, and 8033c, respectively (means ± standard deviations from protein determinations in 10 different OMV preparations from each strain). To quantify the amount of EHEC-Hly in OMVs, 9 µl of TA50 and 8033 OMVs and their serial dilutions (1∶10 to 1∶640), and 9 µl of the same dilutions of purified free EHEC-Hly from TA50 prepared as described previously [6] (protein concentration 30.5 µg/ml) were separated using 10% sodium dodecylsulfate polyacrylamide gel electrophoresis (SDS-PAGE) and immunoblotted using anti-EHEC-Hly antibody [70] and alkaline-phosphatase-conjugated goat anti-rabbit IgG (Dianova, Hamburg, Germany). Signals were developed using a nitro blue tetrazolium chloride/5-bromo-4-chloro-3′-indolyl phosphate, toluidine salt (NBT/BCIP) substrate (Roche, Mannheim, Germany); signal intensities were determined using densitometry (Quantity One software, BioRad, Munich, Germany). The amounts of EHEC-Hly in TA50 and 8033 OMV preparations (µg/ml) were calculated based on a calibration curve generated from dilutions of the purified EHEC-Hly, expressed as means ± standard deviations from measurements of 10 different batches of each OMV preparation, and recalculated for 1 mg of total OMV protein. To confirm the validity of this system, in particular to determine if separation of OMV-associated EHEC-Hly in SDS-PAGE gel and its transfer to a blotting membrane are similar to those of free EHEC-Hly, we mixed EHEC-Hly-free OMVs from strains TA51 and 8033c (∼5.5 µg of OMV protein identical to the protein amounts in 9 µl of OMVs TA50 and 8033) with 3 µg/ml of free EHEC-Hly (the amount of EHEC-Hly identified in TA50 and 8033 OMVs) and incubated the mixtures for 16 h (37°C, 180 rpm) during which time a complete binding of EHEC-Hly to OMVs was expected [16]. The samples were then ultracentrifuged (235,000× g, 2 h, 4°C) and the pellets (TA51 and 8033c OMVs enriched with free EHEC-Hly) were tested for their EHEC-Hly contents in parallel with TA50 and 8033 OMVs as described above, and the EHEC-Hly amounts in the OMVs were determined based on the calibration curve of free EHEC-Hly (a representative experiment is shown in Figure S1A). The absence of any residual free EHEC-Hly in supernatants of EHEC-Hly-enriched TA51 and 8033c OMVs after ultracentrifugation was confirmed using immunoblotting with anti-EHEC-Hly antibody, demonstrating that the entire amount of free EHEC-Hly added bound to OMVs during 16 h.

### Kinetics of OMV and EHEC-Hly production and EHEC-Hly-OMV association

Strains were grown (37°C, 180 rpm) for 24 h in 1 l of LB broth and at each time point of 1.5 h, 3 h, 4.5 h, each hour between 6 h and 15 h, and 24 h a 50 ml aliquot of each culture was collected, bacteria were removed by centrifugation and supernatants were sterile filtered as described above. OMVs were isolated from the filtered supernatants using ultracentrifugation (235,000× g, 2 h, 4°C) and OMV pellets were resuspended in 100 µl of 20 mM TRIS-HCl (pH 8.0); proteins from supernatants after ultracentrifugation were precipitated with 10% trichloroacetic acid (TCA) and resuspended in 100 µl of 20 mM TRIS-HCl (pH 8.0). Aliquots (9 µl) of OMV fractions and TCA-precipitated supernatants were separated by SDS-PAGE, immunoblotted using anti-EHEC-Hly antibody [70] to detect EHEC-Hly and anti-OmpA antibody (kindly provided by Roland Lloubes, Marseille, France) to detect OMVs, and signals were quantified densitometrically as described above. The proportion of EHEC-Hly associated with OMVs at each time interval was calculated as the percentage of densitometric signal of OMV-associated EHEC-Hly from the total EHEC-Hly signal (i.e. the sum of OMV-associated and free EHEC-Hly signals). Bacterial growth was monitored by measuring the optical density of the cultures at 600 nm (OD_600_) (Ultrospec 2100pro Classic, GE Healthcare Europe, Freiburg, Germany) at each time.

### OMVs fractionation and immunoblot analysis of the fractions

Fractionation of OMV preparations was performed by density gradient centrifugation [17], [18]. Briefly, isolated OMVs were resuspended in 20 mM TRIS-HCl (pH 8.0), transferred to the bottom of a 13.2 ml ultracentrifugation tube (Beckman Coulter) and adjusted to 45% OptiPrep (Sigma-Aldrich, Taufkirchen, Germany) in a final volume of 2 ml. Different OptiPrep/HEPES (4-(2-hydroxyethyl)-1-piperazineethanesulfonic acid) layers were sequentially added as follows: 2 ml of 40%, 2 ml of 35%, 3 ml of 30%, 2 ml of 25% and 1 ml of 20%. Gradients were centrifuged (288,000× g, 16 h, 4°C) in a SW 41 Ti rotor (Beckman Coulter) and fractions of equal volumes (1 ml) were removed sequentially from the top. Aliquots (9 µl) of the fractions were separated using SDS-PAGE in 10% or 15% gels, and analyzed using immunoblotting with anti-EHEC-Hly antibody [70] or anti-OmpA antibody as described above.

### Dissociation assay

Dissociation assay was performed as described previously [17]-[19]. Briefly, OMVs from strains TA50 and 8033 (∼20 µg of OMV protein) were suspended in 50 mM HEPES buffer and incubated for 1 h on ice in the absence or presence of either 1 M NaCl or 0.1 M Na_2_CO_3_ or 0.8 M urea or 1% SDS. The samples were then ultracentrifuged (235,000× g, 2 h, 4°C) and pellets (OMVs) were resuspended in 30 µl of 20 mM TRIS-HCl (pH 8.0); soluble proteins from supernatants were precipitated with 10% TCA and resuspended in the same TRIS-HCl volume. Nine µl aliquots of the pellet and supernatant fractions were separated using SDS-PAGE and analyzed using immunoblotting with anti-EHEC-Hly antibody.

To determine effect of pH on association of EHEC-Hly with OMVs, OMVs from strains TA50 and 8033 (∼20 µg of OMV protein) were incubated (1 h at 37°C) in 20 mM TRIS-HCl buffer with pH ranging from 8.0 to 2.0. After ultracentrifugation (235,000× g, 2 h, 4°C), both pellets (OMVs) and TCA-precipitated soluble proteins from supernatants were resuspended in 30 µl of 20 mM TRIS-HCl and analyzed for EHEC-Hly by immunoblotting as described above. Signals were quantified densitometrically and the percentage of EHEC-Hly present in the pellet and supernatant fractions at each particular pH was calculated from the total EHEC-Hly signal at each pH.

### Biochemical composition of EHEC-Hly-containing OMVs

Presence of marker proteins of the outer membrane (OmpA), the periplasm (heat shock protease; HtrA), the inner membrane (leader peptidase A; LepA) and the cytosol (cAMP receptor protein; CRP) was determined in OMVs and TA50 whole cell lysate (control) (all ∼5.5 µg of protein/lane) using immunoblotting with the respective antibodies (the anti-HtrA and anti-LepA antibodies were kindly provided by Thomas Aldick, University of Münster; the anti-CRP mouse monoclonal antibody was from NeoClone Biotechnology, Madison, WI, USA). Presence of DNA in OMVs was analysed using the Quant-iT PicoGreen dsDNA Assay Kit (Molecular Probes, Life Technologies, Darmstadt, Germany) according to the manufacturer's instructions. Intact OMVs (20 µg of OMV protein), either untreated or treated with DNase (50 µg/ml) as described [71], and DNase-treated OMVs which were lysed with GES reagent (5 M guanidinium thiocyanate, 100 mM EDTA, 0.5% sarkosyl) [71] after the DNase treatment were used in the assay. Fluorescence was measured using a fluorescent plate reader (FLUOstar OPTIMA; BMG Labtech, Offenburg, Germany) (excitation 480 nm, emission 520 nm). After subtraction of background fluorescence of the reagent diluent (10 mM TRIS-HCl, 1 mM EDTA, pH 7.5) from fluorescence of each OMV sample, the DNA amount in OMVs was determined based on the standard curve constructed using the bacteriophage lambda DNA provided with the kit, as recommended by the manufacturer.

### Electron microscopy

Overnight LB agar cultures of strains TA50, 8033 and TA51 were harvested into PBS, fixed with 2% paraformaldehyde and 0.2% glutaraldehyde in PBS (pH 7.4) and processed using the method of Tokuyasu [72]. Ultrathin (50 nm) frozen sections were cut and immunogold-stained using anti-*E. coli* LPS antibody (recognizing all *E. coli* LPS types) (Biomol, Hamburg, Germany), anti-O103 LPS antibody (kindly provided by A. Fruth, Robert Koch Institute, Wernigerode, Germany) or anti-EHEC-Hly antibody [70] detected with Protein A Gold (Department of Cell Biology, University Medical Center, Utrecht, Netherlands) with gold particles of 15 nm or 10 nm diameter, respectively. In double staining experiments the anti-*E. coli* LPS or the anti-O103 LPS antibody detected with Protein A Gold with gold particles of 15 nm diameter was followed, after extensive washing, by anti-EHEC-Hly antibody and Protein A Gold with gold particles of 10 nm diameter. Staining with Protein A Gold (gold particles 15 nm or 10 nm) alone, without first antibodies was included as a control of specificity of the immunogold staining. The samples were analyzed at 80 kV on a FEI-Tecnai 12 electron microscope (FEI, Eindhoven, The Netherlands). Photographs of selected areas were documented with imaging plates (Ditabis, Pforzheim, Germany).

### Cell cultures

HBMEC [73] were cultured in Endothelial medium (PAA, Pasching, Austria), and Caco-2 cells (human colonic adenocarcinoma cells; German collection of microorganisms and cell cultures, Braunschweig, Germany; ACC 169) were grown in Quantum 286 epithelial medium (PAA). All incubations were performed at 37°C in an atmosphere with 5% CO_2_. HBMEC in passages 19 to 29 and Caco-2 in passages 21 to 30 were used in experiments.

### Hemolysis and cell lysis assays

Hemolytic activity of EHEC-Hly OMVs was determined as described previously [16] using 100 µl of OMV preparations (∼300 ng of OMV-associated EHEC-Hly). Cytolytic effects of EHEC-Hly-containing OMVs (∼300 ng of OMV-associated EHEC-Hly) on HBMEC and Caco-2 cells were investigated by measuring LDH release from cultured cells using CytoTox 96 assay (Promega, Mannheim, Germany) according to the manufacturer's instructions.

### Kinetics of OMVs uptake by HBMEC and Caco-2 cells

OMVs isolated from strains TA50, TA51, 8033, and 8033c (1 mg of OMV protein/ml) were labeled with 1% (v/v) fluorescence dye DiO (Molecular Probes) as described [21]. Cells were grown to confluence in 75 cm^2^ culture flasks, trypsinized and resuspended in cell culture medium. To monitor the kinetics of OMV uptake, 100 µg of DiO-labeled OMVs were added to a suspension of 5×10^6^ HBMEC or Caco-2 cells and incubated for 4 h (37°C, 5% CO_2_). Samples were taken after 5 min, 15 min, 30 min, 1 h, 2 h, 3 h and 4 h, and fluorescence intensities were measured using a FACScan flow cytometer (Becton Dickinson, Heidelberg, Germany). To determine the proportion of internalized OMVs, extracellular OMV-DiO fluorescence was quenched with trypan blue (final concentration of 0.2% (w/v), which does not enter intact cells [74] and thus allows to detect only intracellularly located OMVs. The samples were applied to the cytometer and emitted fluorescence was measured before (total amount of cell-associated OMVs) and after (internalized OMVs) adding trypan blue. The data were analyzed using CellQuest Pro (Becton Dickinson) and expressed as geometric means of fluorescence from 10,000 events. In each measurement, background fluorescence of cells incubated without OMVs was subtracted from that of OMV-treated cells. To confirm results of flow cytometry, HBMEC and Caco-2 cells grown in 8-well chamber slides (ibidi, Planegg/Martinsried, Germany) were incubated with DiO-labeled OMVs (20 µg of OMV protein) for 4 h. Native cells were analyzed for fluorescence using DIC microscopy and CLSM (LSM 510 META microscope, equipped with a Plan-Apochromat 63x/1.4 oil immersion objective; Carl Zeiss, Jena, Germany) before and after quenching with 0.2% trypan blue. Differences between presence of intracellular and extracellular fluorescence signals before and after trypan blue quenching were determined visually.

### Inhibition of OMV uptake using inhibitors of endocytosis

OMVs isolated from strains TA50, 8033, TA51 and 8033c were labeled with rhodamine isothiocyanate B-R18 (Molecular Probes) as described [35]. In the inhibition assay, monolayers of HBMEC or Caco-2 cells grown in 96-well plates with black frames (Greiner Diagnostics, Bahlingen, Germany) were pretreated with inhibitors of endocytosis (dynasore 80 µM, chlorpromazine 15 µg/ml, or filipin III 10 µg/ml) (all from Sigma-Aldrich) for 1 h at 37°C or remained untreated. Rhodamine-labeled OMVs (3 µg of OMV protein) were then added and incubated with cells in the absence (control) or in the presence of each respective inhibitor (4 h, 37°C). Cells were washed with PBS and fluorescence was measured using a fluorescent plate reader (FLUOstar OPTIMA) (excitation 560 nm, emission 590 nm). Fluorescence of cells incubated with rhodamine-labeled OMVs was normalized to fluorescence of labeled OMVs incubated for 4 h without cells. OMV uptake in the presence of each inhibitor was expressed as the percentage of OMV uptake by control, inhibitor-untreated cells (set as 100%). After measuring fluorescence, cells were analyzed microscopically to ensure that none of the inhibitors in the concentrations used caused cell damage.

### Confocal laser scanning immunofluorescence microscopy

HBMEC and Caco-2 cells were grown in 8-well chamber slides (ibidi) until ∼80% confluence and incubated with TA50 or 8033 OMVs (20 µg of OMV protein containing 100 ng of EHEC-Hly), or with TA51 or 8033c OMVs (20 µg of OMV protein) or with 20 mM TRIS-HCl, pH 8.0 (OMV buffer) for different times. To monitor OMV adherence and internalization, cells were incubated with OMVs for 15 min, 30 min, 1 h and 4 h, washed with PBS (Lonza, Verviers, Belgium), fixed with 3.7% paraformaldehyde, quenched with 0.2 M glycine buffer (pH 7.2), and permeabilized using 0.25% Triton X-100 (Sigma-Aldrich). After blocking with 5% bovine serum albumin (Sigma-Aldrich), OMVs were stained with rabbit polyclonal anti-*E. coli* LPS antibody (Biomol) followed by goat anti-rabbit IgG conjugated with Alexa Fluor 488 (Molecular Probes). Filamentous actin was counterstained with phalloidin tetramethyl rhodamine (phalloidin-TRITC) (Sigma Aldrich).

To determine colocalization of OMVs with caveolin and clathrin, cells were incubated with OMVs for 10 min at 37°C and processed as above. OMVs were stained with rabbit anti-*E. coli* LPS antibody (Biomol) and Alexa Fluor 488-conjugated goat anti-rabbit IgG (Molecular Probes). Caveolin and clathrin were stained using anti-caveolin 1 (7C8) (Novus Biological, Littleton, CO, USA) and anti-clathrin (clone 23) (BD Biosciences, Heidelberg, Germany) mouse monoclonal antibody, respectively, and Cy3-conjugated goat anti-mouse IgG (Dianova). In control experiments, cells were incubated for 10 min with TF-488 (20 µg/ml) or CTB-488 (5 µg/ml) (both Molecular Probes) or with OMV buffer and caveolin and clathrin were stained as described above.

To monitor colocalization of OMVs and EHEC-Hly over time, HBMEC and Caco-2 were incubated with TA50 or 8033 OMVs for 1 h, 4 h, 8 h, 12 h, 16 h, 20 h, and 24 h, fixed, quenched with 0.2 M glycine (pH 2.0), permeabilized using 0.25% Triton X-100, and blocked with 5% goat serum (Molecular Probes). OMVs were stained using mouse monoclonal anti-*E. coli* LPS antibody (clone 2D7/1; Abcam, Cambridge, UK) followed by Alexa Fluor 488-conjugated goat anti-mouse IgG (Molecular Probes), and EHEC-Hly was stained using rabbit polyclonal antibody [70] and Cy3-conjugated goat anti-rabbit IgG (Dianova). In control experiments, cells were treated for 24 h with EHEC-Hly-free OMVs (TA51, 8033c) or with OMV buffer and stained as above. To compare behavior of OMV-associated EHEC-Hly with that of free EHEC-Hly, cells were incubated for 24 h with free recombinant EHEC-Hly purified from strain TA50 [6] in the dose of 10 hemolytic units per ml (corresponding to 0.5 hemolytic dose per well) which does not cause overt cytolysis [6]. EHEC-Hly was stained with anti-EHEC-Hly antibody and Alexa Fluor 488-conjugated goat anti-rabbit IgG (Molecular Probes) and actin with phalloidin-TRITC (Sigma Aldrich).

To investigate colocalization of EHEC-Hly with mitochondria, HBMEC and Caco-2 cells were incubated with TA50 or 8033 OMVs (or with TA51 or 8033c OMVs or no OMVs; controls) for 24 h. MitoTracker Orange CMTMRos (Molecular Probes) (final concentration 100 nM) was added to the cells 45 min before the end of the incubation time. Cells were then washed, fixed, permeabilized, blocked, and stained using anti-EHEC-Hly antibody and Alexa Fluor 488-conjugated goat anti-rabbit IgG.

To determine colocalization of OMVs or EHEC-Hly with endo-lysosomal compartments, cells were incubated with TA50, 8033, TA51 or 8033c OMVs or with OMV buffer for 8 h, 16 h and 24 h. Cells were fixed, quenched with 0.2 M glycine (pH 7.2), and permeabilized and blocked using PBS containing 5% goat serum, 1% bovine serum albumin and 1 mg/ml saponin (Sigma-Aldrich). OMVs were stained with rabbit anti-*E. coli* LPS antibody and Alexa Fluor 488-conjugated goat anti-rabbit IgG. EHEC-Hly was stained using rabbit anti-EHEC-Hly antibody and Alexa Fluor 488-conjugated goat anti-rabbit IgG. Lysosomes were detected either with anti-human LAMP3/CD63 mouse monoclonal antibody (clone H5C6, obtained from the Developmental Studies Hybridoma Bank University of Iowa, Iowa City, IA, USA) and Cy3-conjugated goat anti-mouse IgG (Dianova) or using Lysotracker Red DND-99 (Molecular Probes) (300 nM, 3 h). In some experiments cells were pretreated (1 h, 37°C) with 100 nM bafilomycin A1 (Sigma-Aldrich) before adding OMVs.

For studying colocalization of EHEC-Hly with endoplasmic reticulum and Golgi complex HBMEC and Caco-2 cells were incubated with EHEC-Hly-containing (TA50, 8033) or EHEC-Hly-free (TA51, 8033c) OMVs or with OMV buffer for 8 h, 16 h, and 24 h. Endoplasmic reticulum was stained using anti-Protein Disulfide Isomerase (PDI) mouse monoclonal antibody (clone C-2) and Golgi complex using anti-GM130 mouse monoclonal antibody (clone (B-10) (both from Santa Cruz Biotechnology, Inc., Heidelberg, Germany) and Cy3-conjugated goat anti-mouse IgG (Dianova). EHEC-Hly was stained with anti-EHEC-Hly antibody and Alexa Fluor 488-conjugated goat anti-rabbit IgG (Molecular Probes).

Nuclei were stained in all protocols using 5 µM DRAQ5 (1,5-bis{[2-(di-methylamino) ethyl]amino}-4, 8-dihydroxyanthracene-9,10-dione) (Cell Signaling; distributed by New England Biolabs, Frankfurt am Main, Germany). Slides were mounted in Dako fluorescence mounting medium (Dako, Hamburg, Germany) and all preparations (with the exception mentioned below) were analyzed using a confocal laser-scanning microscope (LSM 510 META microscope, equipped with a Plan-Apochromat 63x/1.4 oil immersion objective; Carl Zeiss). To quantify colocalizations of various fluorescence signals, digital colocalization images were imported into BioImageXD6 [75], and signals were manually thresholded. Subsequently, the percentage of colocalized signals in at least five different samples was calculated using the BioImageXD6 colocalization tool. Statistical significance of the results was evaluated using the paired Student's *t*-test. Preparations showing interaction of free EHEC-Hly with cells were analyzed using a fluorescence microscope (Axio Imager.A1, Carl Zeiss) and photographed with an AxioCam CCD camera (Carl Zeiss).

### Detection of OMVs and EHEC-Hly in isolated lysosomal and mitochondrial fractions from HBMEC and Caco-2 cells

HBMEC and Caco-2 cells grown in 75 cm^2^ culture flasks until confluence were incubated with TA50 or 8033 OMVs (100 µg of OMV protein containing 500 ng of EHEC-Hly) for 8 h, 16 h and 24 h or remained untreated (control). In some experiments, cells were pretreated with 100 nM bafilomycin A1 (Sigma-Aldrich) for 1 h at 37°C prior to adding OMVs. Lysosomal and mitochondrial fractions were isolated using the Lysosome Enrichment Kit for Tissue and Cultured Cells and the Mitochondria Isolation Kit for Cultured Cells (both from Thermo Scientific, Limburg, Germany), respectively, according to the manufacturer's instructions. Briefly, to isolate lysosomal fractions, ∼250 mg of HBMEC or Caco-2 cells harvested by trypsinization were washed with PBS, treated with Lysosome Enrichment Reagent A supplemented with a protease inhibitor cocktail (Complete Mini, EDTA-free protease inhibitor cocktail tablets; Roche) for 2 min on ice, and lysed in a 2-ml Dounce tissue grinder (Sigma-Aldrich) using 50 strokes for HBMEC and 80 strokes for Caco-2 cells. After adding Lysosome Enrichment Reagent B (with a protease inhibitor cocktail) to lysed cells, samples were centrifuged (500× g, 10 min, 4°C), supernatants were adjusted with the OptiPrep Cell Separation Media to a final concentration of 15% and loaded on discontinuous Optiprep gradients (30%, 27%, 23%, 20% and 17%) in 13.2 ml ultracentrifugation tubes (Beckman Coulter). The tubes were centrifuged (145,000× g, 2 h, 4°C) in a SW 41 Ti rotor (Beckman Coulter) and the lysosomal fractions located at the top of the gradients were collected and washed with PBS (18,000× g, 30 min, 4°C) to remove Optiprep. Lysosomal pellets were then once more washed with Gradient Dilution Buffer (18,000× g, 30 min, 4°C).

To isolate mitochondria, ∼5×10^7^ cells harvested by trypsinization and washed with PBS were treated (2 min on ice) with Reagent A from the Mitochondria Isolation Kit (with a protease inhibitor cocktail) and lysed using a Dounce tissue grinder as described above. Reagent C (with a protease inhibitor cocktail) was added to lysed cells and the samples were centrifuged (700× g, 10 min, 4°C) to remove nuclei and cell debris. Supernatants were transferred to fresh tubes and centrifuged (3000× g, 15 min, 4°C) to collect mitochondria, which were then washed with Reagent C (12,000× g, 5 min, 4°C).

The isolated lysosomal and mitochondrial fractions were resuspended in 20 mM TRIS-HCl (pH 8.0) and protein concentration was determined using the Roti-Nanoquant reagent (Carl Roth). For immunoblot analysis, the samples were boiled with SDS-PAGE sample buffer, separated (50 µg of protein/lane) using SDS-PAGE and immunoblotted with antibodies against OmpA, EHEC-Hly, LAMP-1 (Cell Signaling), porin-2 (Novus Biological) or LC3B (Cell Signaling) and alkaline-phosphatase-conjugated goat anti-rabbit IgG (Dianova). Signals were developed using a NBT/BCIP substrate (Roche) and signal intensities were determined using densitometry (Quantity One, BioRad).

### Detection of cytochrome c in mitochondrial and cytosolic fractions

HBMEC and Caco-2 cells grown in 12-well plates (∼2×10^6^ cells) were incubated with TA50 or 8033 OMVs (20 µg of OMV protein containing 100 ng of EHEC-Hly) or with the corresponding protein amounts of EHEC-Hly-negative OMVs (TA51 or 8033c) for 8 h, 16 h, and 24 h and mitochondrial and cytosolic fractions were prepared using the Cytochrome c Releasing Apoptosis Assay Kit (BioVision, Heidelberg, Germany) according to the manufacturer's instructions. Aliquots of the mitochondrial and cytosolic fractions (50 µg protein/lane) were separated using 15% SDS-PAGE and probed with antibody against cytochrome c (provided with the kit) and goat anti-mouse horseradish peroxidase-conjugated IgG (Cell Signaling). Signals were developed using a chemiluminescence enhancement kit (SuperSignal West Pico; Thermo Scientific, Bonn, Germany) and visualized with a chemiluminescence imager and a CCD camera (Chemi Doc XRS, Bio-Rad). The membranes were stripped (Restore Western blot stripping buffer; Thermo Scientific) and reprobed with antibodies against the mitochondrial marker porin-2 (rabbit polyclonal antibody, Novus Biological) or the cytosolic marker actin (mouse monoclonal antibody, Santa Cruz Biotechnology) (used as loading controls) and horseradish peroxidase-conjugated secondary antibodies (Cell Signaling).

### Determination of mitochondrial transmembrane potential

HBMEC and Caco-2 cells grown in 12-well plates (∼2×10^6^ cells) were incubated with EHEC-Hly-containing OMVs (20 µg of OMV protein containing 100 ng of EHEC-Hly) or with the corresponding amounts of EHEC-Hly-negative OMVs for 8 h, 16 h and 24 h. Cells were stained with 100 nM TMRE (Molecular Probes) for 20 min, trypsinized and staining was quantified by flow cytometry (FACScalibur; Becton Dickinson) using red emission (FL-2 channel, 575 nm). Median fluorescence values of OMV-treated cells were expressed as the percentage of the median fluorescence of untreated cells (defined as 100%). Valinomycin (100 nM, 15 min) (Sigma-Aldrich) served as a positive control.

### Caspase-9, caspase-3, and caspase-8 activity assays and PARP cleavage

Caspase-9, caspase-3 and caspase-8 activity was assayed using the Caspase Colorimetric Substrate Kit I (GenTex; distributed by Biozol Diagnostica, Eching, Germany). HBMEC and Caco-2 cells grown to confluence in 12-well plates were treated with EHEC-Hly-containing OMVs (TA50 or 8033) (20 µg or 10 µg of OMV protein containing 100 ng or 50 ng of OMV-associated EHEC-Hly, respectively) or with EHEC-Hly-negative OMVs (TA51 or 8033c) (20 µg OMV protein) for 8 h, 24 h, and 48 h. Untreated cells cultured for the times indicated in cell culture medium served as controls. Cells were trypsinized, collected by centrifugation, and the pellets from 2×10^6^ cells were lysed using lysis buffer (10 min on ice). Cell lysates were centrifuged (10,000× g, 5 min) and colorimetric substrates for caspase-9 (Ac-LEHD-pNA), caspase-3 (Ac-DEVD-pNA), or caspase-8 (Ac-IETD-pNA) were added (final concentration 200 µM) to the supernatants together with the reaction buffer. After incubation for 90 min at 37°C, the color intensity, which is directly proportional to the level of caspase enzymatic activity, was measured spectrophotometrically at 405 nm (Microplate Reader, Dynex Technologies, Chantilly, Va., USA). The activity of each caspase in OMV-treated cells was expressed as a fold-increase of its activity in untreated cells (defined as 1). In some experiments, cells were pretreated with inhibitors of caspase-9 (z-LEHD-fmk) or caspase-3 (z-DEVD-fmk) (both 100 µM) or with the pan-caspase inhibitor z-VAD-fmk (50 µM) (all from R & D Systems, Wiesbaden, Germany) for 30 min before adding the probes.

To detected PARP cleavage, cell lysates prepared as above were separated using SDS-PAGE (10% gel, 50 µg protein/lane), immunoblotted using anti-PARP antibody and horseradish peroxidase-labeled goat anti-rabbit IgG (Cell Signaling), developed using the chemiluminescence enhancement kit (Thermo Scientific) and visualized with a chemiluminescence imager as described above. After stripping, the membranes were reprobed with antibody against actin (Santa Cruz Biotechnology) and horseradish peroxidase-labeled goat anti-mouse IgG (Cell Signaling). Cells exposed to 1 µM staurosporin (Sigma-Aldrich) for 3 h were a positive control.

### Detection of DNA laddering

DNA laddering was assayed using the Apoptotic DNA Ladder Kit (Roche) using modifications of the manufacturer's instructions. Briefly, ∼2×10^6^ HBMEC or Caco-2 cells grown in 12-well plates were exposed to EHEC-Hly-containing OMVs (TA50 or 8033, 20 µg of OMV protein containing 100 ng of EHEC-Hly) or to EHEC-Hly-negative OMVs (TA51 or 8033c, 20 µg of OMV protein) or to 1 µM staurosporin (positive control) for 48 h, or remained untreated (negative control). Cells were lysed with lysis buffer, the lysates were centrifuged through DNA binding columns and DNA was eluted with 10 mM Tris (pH 8.5). The eluates were centrifuged (13,000× g, 10 min) to separate fragmented from intact DNA. Supernatants containing fragmented DNA were 10-fold concentrated using a speed vacuum concentrator (Eppendorf, Hamburg, Germany) and treated with RNase (Sigma-Aldrich) (2 µg/ml, 30 min, 37°C). Equal amounts of the DNA (1.5 µg) were separated by agarose gel electrophoresis (1.2% w/v), gels were stained with Midori Green Advance (Biozym Scientific, Hessisch Oldendorf, Germany) and photographed (Gel Stick Imager, INTAS Science Imaging Instruments, Göttingen, Germany).

### TUNEL assay

TUNEL assay was performed using the In Situ Cell Death Detection Kit (Roche). HBMEC and Caco-2 cells grown until ∼80% confluence in 8-well permanox Lab-Tek chamber slides (Nunc, Langenselbold, Germany) were incubated for 8 h, 24 h and 48 h with EHEC-Hly-containing OMVs (TA50 or 8033; 20 µg of OMV protein containing 100 ng of EHEC-Hly) or with the corresponding protein amounts of EHEC-Hly-free OMVs (TA51 or 8033c), or with 1 µM staurosporin (positive control) or remained untreated (negative control). If required, cells were pretreated with the pan-caspase inhibitor z-VAD-fmk (50 µM) 30 min before adding the probes. Cells were washed, fixed, permeabilized and stained with the TUNEL reaction mixture according to the manufacturer's instructions. Actin and nuclei were counterstained with phalloidin-TRITC and DAPI (4′,6-diamidino-2-phenylindol) (both (Sigma-Aldrich), respectively. The cells were examined using a fluorescence microscope (Axio Imager.A1, Carl Zeiss) and the percentage of TUNEL-positive nuclei (from total of 200-300 cells analyzed) was calculated.

### Statistical analysis

Statistical analysis was performed using the paired or unpaired Student's *t*-test; *p*<0.05 was considered significant.

### Accession numbers for EHEC hemolysin

GenBank X86087.1: EHEC hemolysin operon; *hlyC* gene; *hlyA* gene; *hlyB* gene; *hlyD* gene

UniProtKB/Swiss-Prot: Q47460: EHEC-hlyC protein

UniProtKB/Swiss-Prot: Q47461: EHEC-hlyA protein

UniProtKB/Swiss-Prot: Q46717: EHEC-hlyB protein

UniProtKB/Swiss-Prot: Q47463: EHEC-hlyD protein

## Supporting Information

Figure S1 **Characterization of EHEC-Hly-containing OMVs.** (**A**) Quantification of EHEC-Hly in OMVs. Nine µl of serial dilutions of purified recombinant free EHEC-Hly from strain TA50 (protein concentration 30.5 µg/ml; EHEC-Hly concentrations in serial dilutions [µg/ml] are shown below the blot), and 9 µl of EHEC-Hly-containing (TA50, 8033) and EHEC-Hly-free (TA51, 8033c) OMVs (the latter two before and after enrichment with 3 µg/ml of free EHEC-Hly as described in Materials and Methods) were separated using SDS-PAGE, transferred to a membrane and immunoblotted with anti-EHEC-Hly antibody. Signals were quantified densitometrically (graph below the blot) and the concentration of EHEC-Hly in OMVs (µg/ml; shown below the blot) was calculated based on a calibration curve generated from dilutions of free EHEC-Hly and recalculated for 1 mg of total OMV protein. A representative experiment is shown in which TA50 and 8033 OMVs were determined to contain 3.03 µg/ml and 3.01 µg/ml of EHEC-Hly corresponding to 5.1 µg and 4.9 µg of EHEC-Hly per 1 mg of OMV protein, respectively. EHEC-Hly-free TA51 and 8033c OMVs enriched before the analysis with 3 µg/ml of free EHEC-Hly (the EHEC-Hly amount present in OMVs TA50 and 8033) were determined to contain 3.01 µg/ml and 3.0 µg/ml of EHEC-Hly corresponding to 4.9 µg and 4.8 µg of EHEC-Hly per 1 mg of OMV protein, respectively. This experiment thus demonstrated that OMV-associated EHEC-Hly separates during SDS-PAGE and is transferred to a blotting membrane equally to free EHEC-Hly and proved the validity of the data on EHEC-Hly content in TA50 and 8033 OMVs based on calibration curve of free EHEC-Hly. (**B**) EHEC-Hly co-fractionates with OMVs. TA50 and 8033 OMVs were fractionated using OptiPrep density gradient (see Materials and Methods), 9 µl aliquots of each fraction were separated using SDS-PAGE and immunoblotted with antibodies against OmpA (an OMV marker) or EHEC-Hly. The numbers above the blots indicate the order of the OptiPrep fractions in which they were collected from the top to the bottom. The lanes designated OMV contain 9 µl of non-fractionated OMVs. (**C**) EHEC-Hly is tightly associated with OMVs (dissociation assay). TA50 and 8033 OMVs were incubated (1 h on ice) in HEPES buffer alone (buffer control), or in HEPES buffer containing 1 M NaCl or 0.1 M Na_2_CO_3_ or 0.8 M urea or 1% SDS. After ultracentrifugation, OMV-containing pellets (P) and TCA-precipitated supernatants (S) (9 µl each) were separated electrophoretically and immunoblotted with anti-EHEC-Hly antibody. (**D**) Biochemical composition of OMVs. TA50 and 8033 OMVs and TA50 whole bacterial lysate (WCL; control) (∼5.5 µg protein/lane) were SDS-PAGE-separated and analyzed using immunoblotting with antibodies against OmpA (the outer membrane marker), HtrA (heat shock protease; periplasmic marker), LepA (leader peptidase A; the inner membrane marker) and cAMP receptor protein (CRP; cytosol marker). Sizes of immunoreactive bands in panels A–D are indicated along the right side of the blots. (**E**) EHEC-Hly-containing OMVs cause hemolysis but fail to lyse HBMEC and Caco-2 cells. OMVs from strains TA50 and 8033 (100 µl containing 300 ng of EHEC-Hly) or from EHEC-Hly-negative strains TA51 and 8033c were incubated with washed human erythrocytes or with HBMEC or Caco-2 cells for 48 h in the presence of 10 mM CaCl_2_. Hemolysis and cell lysis (the latter measured by LDH release using the CytoTox 96 assay) were detected in 4 h intervals. Data are presented as means ± standard deviations from three independent experiments.
(TIF)

Figure S2 **OMVs are internalized by HBMEC and Caco-2 cells.** HBMEC and Caco-2 monolayers were incubated with DiO-labeled OMVs from strains TA50, TA51, 8033 or 8033c (20 µg of OMV protein) for 4 h. Native cells were analyzed for fluorescence using DIC microscopy and CLSM before (total cell-associated and extracellular OMVs) and after (internalized OMVs) trypan blue quenching. Note that DiO-labeled OMVs located outside cells (examples indicated by arrows) or within damaged cells (indicated by an arrow with asterisk) are quenched, whereas those located within intact cell bodies are not quenched, demonstrating their internalization.
(TIF)

Figure S3 **Controls of secondary antibodies.** (**A, B**) HBMEC and Caco-2 cells were incubated with EHEC-Hly-containing (TA50 or 8033) or EHEC-Hly-free (TA51 or 8033c) OMVs for 24 h. Cells were fixed, permeabilized and stained with Cy3-conjugated goat anti-mouse IgG and Alexa Fluor 488-conjugated goat anti-rabbit IgG (**A**) or with Cy3-conjugated goat anti-rabbit IgG and Alexa Fluor 488-conjugated goat anti-mouse IgG (**B**) in the absence of primary antibodies. Nuclei were stained with DRAQ5. Scale bars are 10 µm.
(TIF)

Figure S4 **EHEC-Hly internalized via OMVs separates from OMVs during intracellular trafficking which does not involve endoplasmic reticulum and Golgi complex.** (**A**) HBMEC and Caco-2 cells were incubated with TA50 or 8033 OMVs for the times indicated and analyzed for OMVs and EHEC-Hly using CLSM as described in legend to Figure 5. Digital colocalization images were imported into BioImageXD6 software and the percentage of colocalization between OMVs and EHEC-Hly at each time (average from at least five different samples) was calculated using the BioImageXD6 colocalization tool. * Significant differences between incubation times (*p*<0.05; paired Student's *t*-test). (**B**) Free EHEC-Hly is not internalized by HBMEC and Caco-2 cells. Cells were incubated for 24 h with a sublytic dose (10 hemolytic units per ml) of free recombinant EHEC-Hly from strain TA50 or remained untreated (control). EHEC-Hly (EHly) was stained with rabbit anti-EHEC-Hly antibody and Alexa Fluor 488-conjugated goat anti-rabbit IgG (green), actin with phalloidin-TRITC (red) and nuclei with DRAQ5 (blue). (**C, D**) HBMEC and Caco-2 cells were incubated with EHEC-Hly-containing (TA50, 8033) or EHEC-Hly-free (TA51, 8033c) OMVs or with OMV buffer instead of OMVs for 16 h. Endoplasmic reticulum (**C**) and Golgi complex (**D**) were stained using mouse anti-PDI and anti-GM130 antibody, respectively, and Cy3-conjugated goat anti-mouse IgG (red) and EHEC-Hly (EHly) with rabbit anti-EHEC-Hly antibody and Alexa Fluor 488-conjugated goat anti-rabbit IgG (green). Nuclei were stained with DRAQ5. Results shown are also representative of time points of 8 h and 24 h. Note that the seeming partial colocalization of EHEC-Hly and ER in HBMEC and Caco-2 cells exposed to TA50 or 8033 OMVs results from the overlapping strong PDI signal in the perinuclear regions. A structural colocalization was never observed.
(TIF)

Figure S5 **Colocalization of OMVs and EHEC-Hly with endo-lysosomal compartments detected with anti-CD63 antibody.** (**A**) HBMEC and Caco-2 cells were incubated with EHEC-Hly-containing (TA50 or 8033) or EHEC-Hly-free (TA51 or 8033c) OMVs for 16 h. OMVs were stained with rabbit anti-*E. coli* LPS antibody and Alexa Fluor 488-conjugated goat anti-rabbit IgG (green), lysosomes with mouse anti-CD63 antibody and Cy3-conjugated goat anti-mouse IgG (red), and nuclei with DRAQ5 (blue). (**B**) HBMEC and Caco-2 cells were incubated with TA50 or 8033 OMVs for 16 h and stained as described above except that in lieu of OMVs, EHEC-Hly (EHly) was detected with rabbit anti-EHEC-Hly antibody and Alexa Fluor 488-conjugated goat anti-rabbit IgG (green). (**C**) HBMEC and Caco-2 cells were incubated for 24 h with 20 mM TRIS-HCl (OMV buffer) instead of OMVs and stained for OMVs or EHEC-Hly as described in (**A**) and (**B**). Pictures were taken using a laser-scanning microscope (LSM 510 META microscope, equipped with a Plan-Apochromat 63x/1.4 oil immersion objective). All three fluorescence images were merged and consisted of one optical section of a z-series with a pinhole of 1 airy unit. Colocalized red and green signals appear in yellow (examples are depicted by arrows). Scale bars are 10 µm. The percentages of colocalizations between OMVs and CD63-positive compartments (**A**) and EHEC-Hly and CD63-positive compartments (**B**) were calculated using BioImageXD6 colocalization tool and are indicated (averages from at least five different samples) by white numbers in the respective panels. (**D, E**) Graphical presentation of colocalizations between OMVs and CD63-positive compartments (**D**) and EHEC-Hly and CD63-positive compartments (**E**) during time (8 h, 16 h, 24 h) calculated using BioImageXD6 colocalization tool. * Significant differences between incubation times (*p*<0.05; paired Student's *t*-test).
(TIF)

Figure S6 **Releasing of EHEC-Hly from lysosomes leads to a transient loss of lysosomal function.** (**A**) HBMEC and Caco-2 cells were incubated with EHEC-Hly-containing (TA50 or 8033) or EHEC-Hly-free (TA51 or 8033c) OMVs for 16 h. OMVs were stained with rabbit anti-*E. coli* LPS antibody and Alexa Fluor 488-conjugated goat anti-rabbit IgG (green), lysosomes with Lysotracker Red DND-99 (Lyso) (red) and nuclei with DRAQ5 (blue). (**B**) HBMEC and Caco-2 cells were incubated with TA50 or 8033 OMVs for 16 h and stained as described above, except that in lieu of OMVs, EHEC-Hly (EHly) was detected with rabbit anti-EHEC-Hly antibody and Alexa Fluor 488-conjugated goat anti-rabbit IgG (green). Pictures were taken and processed as described in the legend to Figure S5. Colocalized red and green signals appear in yellow (examples depicted by arrows). The percentages of colocalizations between OMVs and Lysotracker Red DND-99 (**A**) and EHEC-Hly and Lysotracker Red DND-99 (**B**) were calculated using BioImageXD6 colocalization tool and are indicated (averages from at least five different samples) by white numbers. (**C**) Graphical presentation of colocalizations between OMVs and Lysotracker Red DND-99 during time calculated using BioImageXD6 colocalization tool. * Significant differences between OMVs/incubation times (*p*<0.05; paired Student's *t*-test). (**D, E**) HBMEC and Caco-2 cells were treated with OMV buffer instead of OMVs and stained for OMVs (**D**) or EHEC-Hly (**E**) and lysosomes as described in (A) and (B). (**F**) Lysosomes in control, OMV-untreated cells were double-stained using anti-CD63 antibody and Alexa Fluor 488-conjugated goat anti-mouse IgG (green) and Lysotracker Red DND-99 (red); nuclei were stained with DRAQ5 (blue). Single channels and their merge are shown; colocalized red and green signals appear in yellow (examples depicted by arrows). Scale bars in all panels are 10 µm.
(TIF)

Figure S7 **EHEC-Hly does not activate caspase-8.** HBMEC (**A**) and Caco-2 cells (**B**) were incubated with EHEC-Hly-containing OMVs from strains TA50 or 8033 (20 µg or 10 µg of OMV protein containing 100 ng or 50 ng of EHEC-Hly, respectively) or with EHEC-Hly-free OMVs from strains TA51 or 8033c (20 µg of OMV protein) for the times indicated or remained untreated. Cells were lysed, the lysates were incubated with the colorimetric substrate of caspase-8 (Ac-IETD-pNA) and the color intensity, which is proportional to the level of caspase-8 enzymatic activity, was measured spectrophotometrically. The caspase-8 activity in OMV-treated cells was expressed as a fold-increase of that in untreated control cells (defined as 1). Data are means ± standard deviations from three independent experiments.
(TIF)

Table S1 **Presence of DNA in OMVs.**
(DOC)

Table S2 **Percentages of TA50, 8033, TA51 and 8033c OMVs internalized over time by HBMEC and Caco-2 cells.**
(DOC)
