# Supplementary figures and images for: Enterohemorrhagic Escherichia coli Hemolysin Employs Outer Membrane Vesicles to Target Mitochondria and Cause Endothelial and Epithelial Apoptosis
Source: PLoS Pathog. 2013 Dec 12;9(12):e1003797. doi: 10.1371/journal.ppat.1003797 (PMC3861543; doi:10.1371/journal.ppat.1003797)

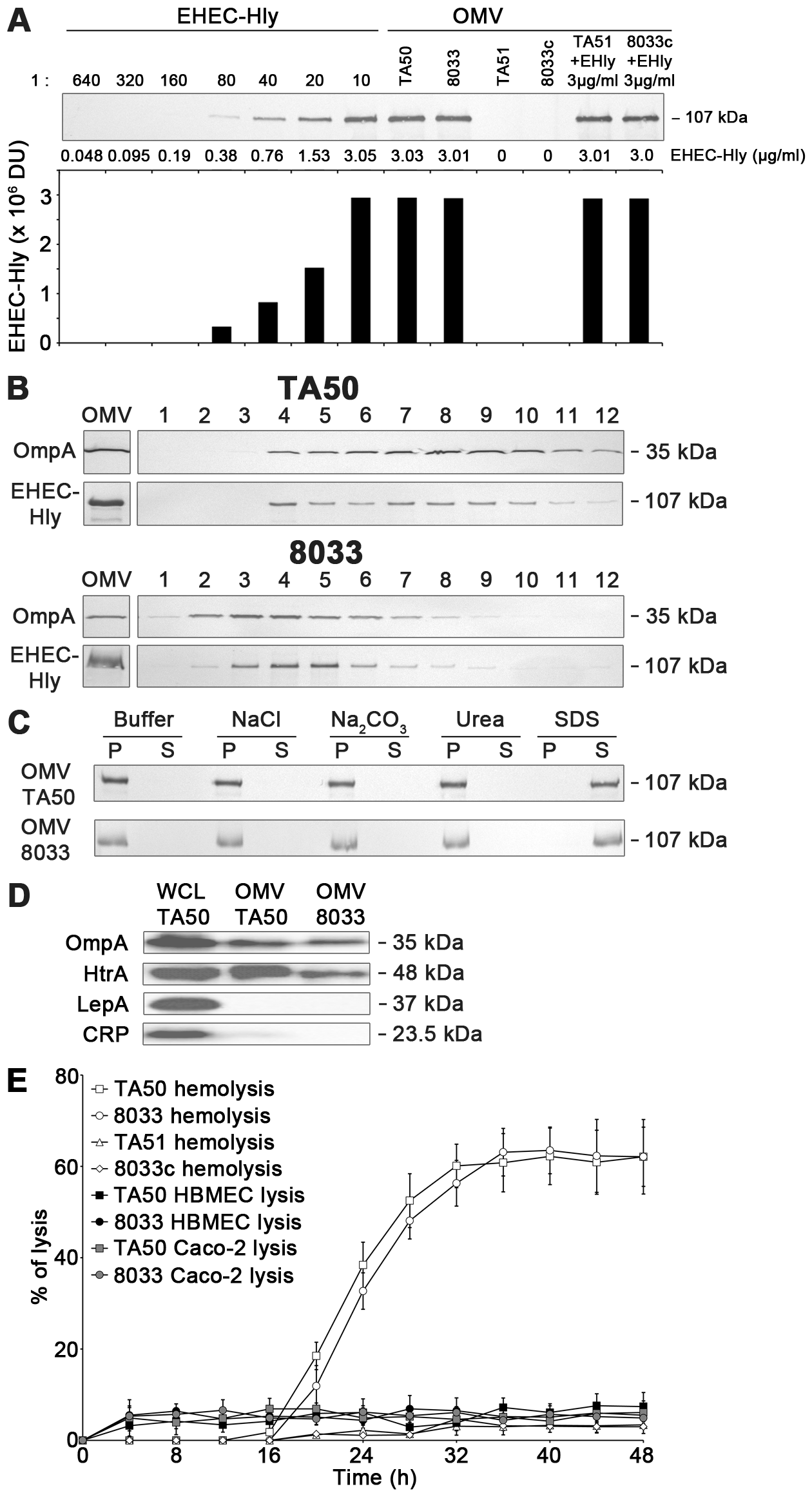

Supplement: Figure S1 — Characterization of EHEC-Hly-containing OMVs. (A) Quantification of EHEC-Hly in OMVs. Nine µl of serial dilutions of purified recombinant free EHEC-Hly from strain TA50 (protein concentration 30.5 µg/ml; EHEC-Hly concentrations in serial dilutions [µg/ml] are shown below the blot), and 9 µl of EHEC-Hly-containing (TA50, 8033) and EHEC-Hly-free (TA51, 8033c) OMVs (the latter two before and after enrichment with 3 µg/ml of free EHEC-Hly as described in Materials and Methods) were separated using SDS-PAGE, transferred to a membrane and immunoblotted with anti-EHEC-Hly antibody. Signals were quantified densitometrically (graph below the blot) and the concentration of EHEC-Hly in OMVs (µg/ml; shown below the blot) was calculated based on a calibration curve generated from dilutions of free EHEC-Hly and recalculated for 1 mg of total OMV protein. A representative experiment is shown in which TA50 and 8033 OMVs were determined to contain 3.03 µg/ml and 3.01 µg/ml of EHEC-Hly corresponding to 5.1 µg and 4.9 µg of EHEC-Hly per 1 mg of OMV protein, respectively. EHEC-Hly-free TA51 and 8033c OMVs enriched before the analysis with 3 µg/ml of free EHEC-Hly (the EHEC-Hly amount present in OMVs TA50 and 8033) were determined to contain 3.01 µg/ml and 3.0 µg/ml of EHEC-Hly corresponding to 4.9 µg and 4.8 µg of EHEC-Hly per 1 mg of OMV protein, respectively. This experiment thus demonstrated that OMV-associated EHEC-Hly separates during SDS-PAGE and is transferred to a blotting membrane equally to free EHEC-Hly and proved the validity of the data on EHEC-Hly content in TA50 and 8033 OMVs based on calibration curve of free EHEC-Hly. (B) EHEC-Hly co-fractionates with OMVs. TA50 and 8033 OMVs were fractionated using OptiPrep density gradient (see Materials and Methods), 9 µl aliquots of each fraction were separated using SDS-PAGE and immunoblotted with antibodies against OmpA (an OMV marker) or EHEC-Hly. The numbers above the blots indicate the order of the OptiPrep fractions in which [file ppat.1003797.s001.tif]

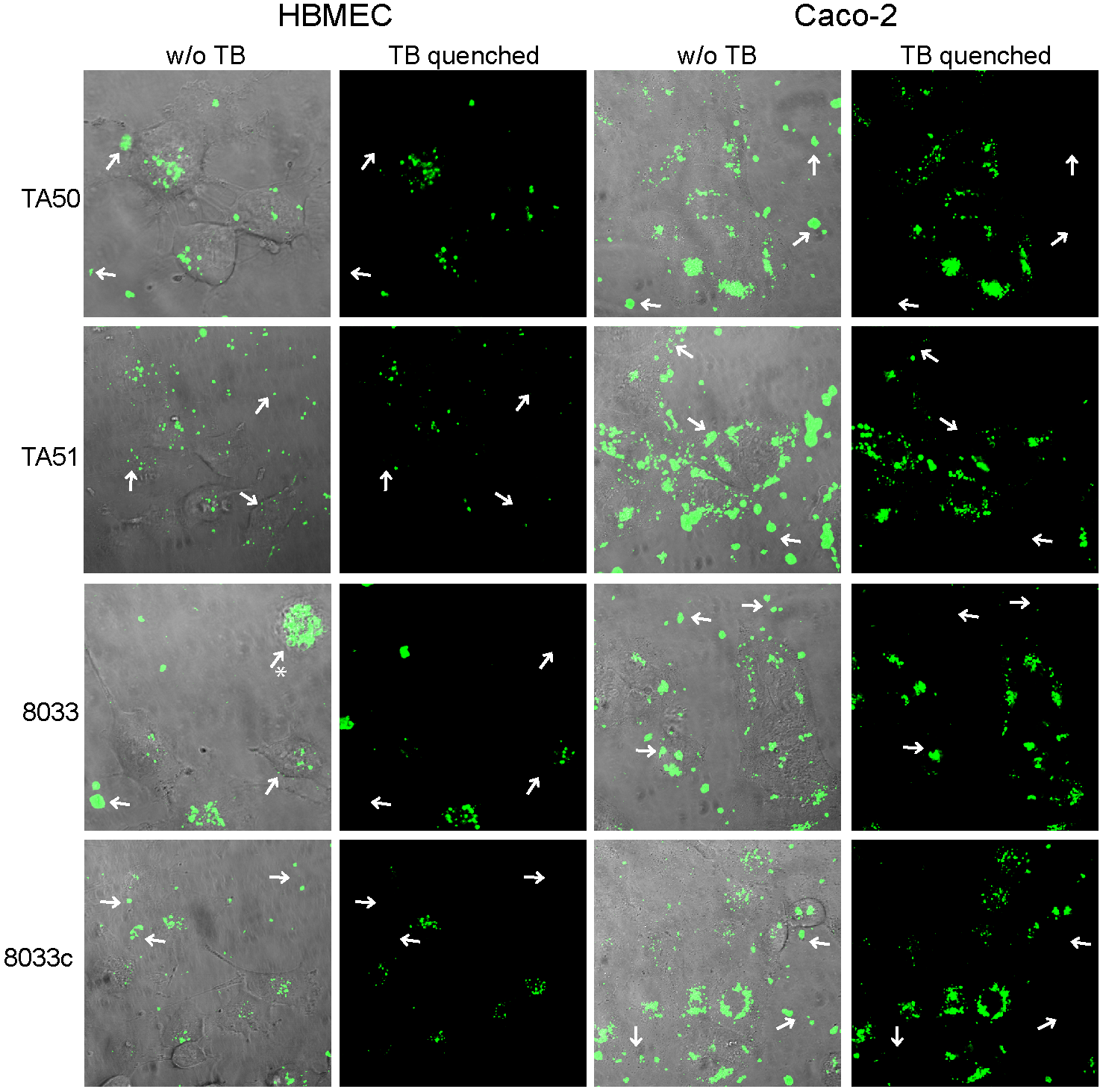

Supplement: Figure S2 — OMVs are internalized by HBMEC and Caco-2 cells. HBMEC and Caco-2 monolayers were incubated with DiO-labeled OMVs from strains TA50, TA51, 8033 or 8033c (20 µg of OMV protein) for 4 h. Native cells were analyzed for fluorescence using DIC microscopy and CLSM before (total cell-associated and extracellular OMVs) and after (internalized OMVs) trypan blue quenching. Note that DiO-labeled OMVs located outside cells (examples indicated by arrows) or within damaged cells (indicated by an arrow with asterisk) are quenched, whereas those located within intact cell bodies are not quenched, demonstrating their internalization. (TIF) [file ppat.1003797.s002.tif]

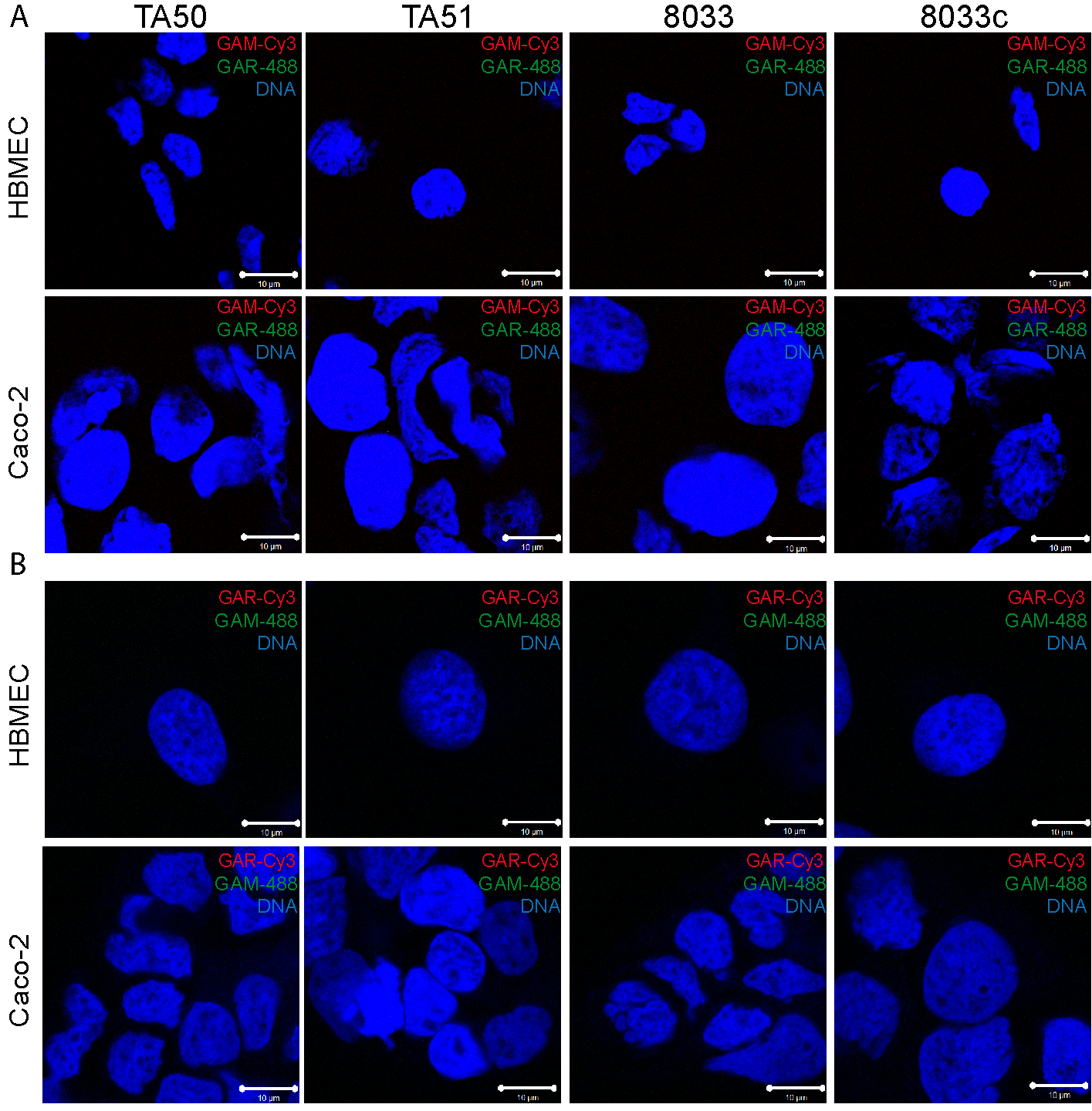

Supplement: Figure S3 — Controls of secondary antibodies. (A, B) HBMEC and Caco-2 cells were incubated with EHEC-Hly-containing (TA50 or 8033) or EHEC-Hly-free (TA51 or 8033c) OMVs for 24 h. Cells were fixed, permeabilized and stained with Cy3-conjugated goat anti-mouse IgG and Alexa Fluor 488-conjugated goat anti-rabbit IgG (A) or with Cy3-conjugated goat anti-rabbit IgG and Alexa Fluor 488-conjugated goat anti-mouse IgG (B) in the absence of primary antibodies. Nuclei were stained with DRAQ5. Scale bars are 10 µm. (TIF) [file ppat.1003797.s003.tif]

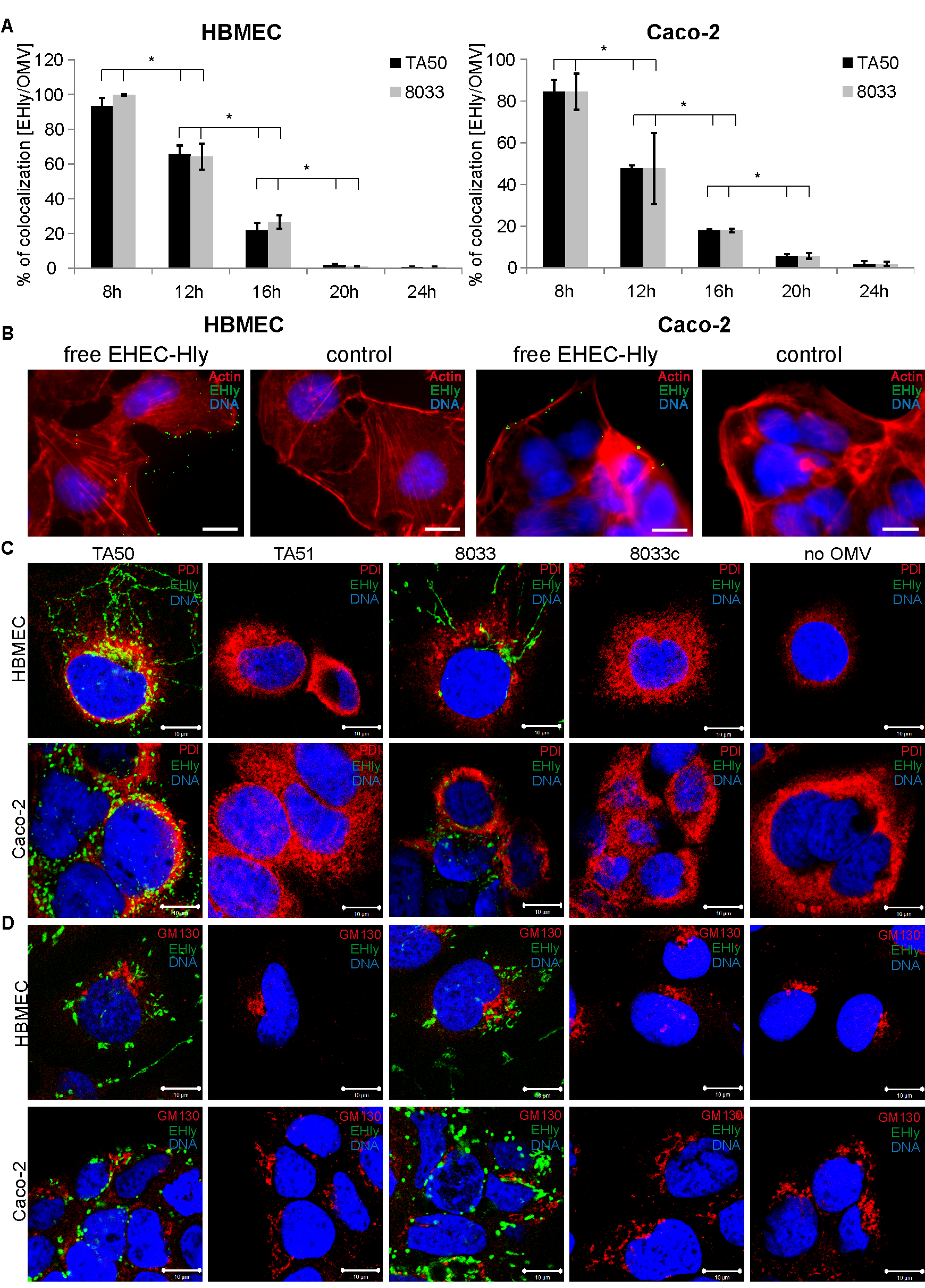

Supplement: Figure S4 — EHEC-Hly internalized via OMVs separates from OMVs during intracellular trafficking which does not involve endoplasmic reticulum and Golgi complex. (A) HBMEC and Caco-2 cells were incubated with TA50 or 8033 OMVs for the times indicated and analyzed for OMVs and EHEC-Hly using CLSM as described in legend to Figure 5. Digital colocalization images were imported into BioImageXD6 software and the percentage of colocalization between OMVs and EHEC-Hly at each time (average from at least five different samples) was calculated using the BioImageXD6 colocalization tool. * Significant differences between incubation times (p<0.05; paired Student's t-test). (B) Free EHEC-Hly is not internalized by HBMEC and Caco-2 cells. Cells were incubated for 24 h with a sublytic dose (10 hemolytic units per ml) of free recombinant EHEC-Hly from strain TA50 or remained untreated (control). EHEC-Hly (EHly) was stained with rabbit anti-EHEC-Hly antibody and Alexa Fluor 488-conjugated goat anti-rabbit IgG (green), actin with phalloidin-TRITC (red) and nuclei with DRAQ5 (blue). (C, D) HBMEC and Caco-2 cells were incubated with EHEC-Hly-containing (TA50, 8033) or EHEC-Hly-free (TA51, 8033c) OMVs or with OMV buffer instead of OMVs for 16 h. Endoplasmic reticulum (C) and Golgi complex (D) were stained using mouse anti-PDI and anti-GM130 antibody, respectively, and Cy3-conjugated goat anti-mouse IgG (red) and EHEC-Hly (EHly) with rabbit anti-EHEC-Hly antibody and Alexa Fluor 488-conjugated goat anti-rabbit IgG (green). Nuclei were stained with DRAQ5. Results shown are also representative of time points of 8 h and 24 h. Note that the seeming partial colocalization of EHEC-Hly and ER in HBMEC and Caco-2 cells exposed to TA50 or 8033 OMVs results from the overlapping strong PDI signal in the perinuclear regions. A structural colocalization was never observed. (TIF) [file ppat.1003797.s004.tif]

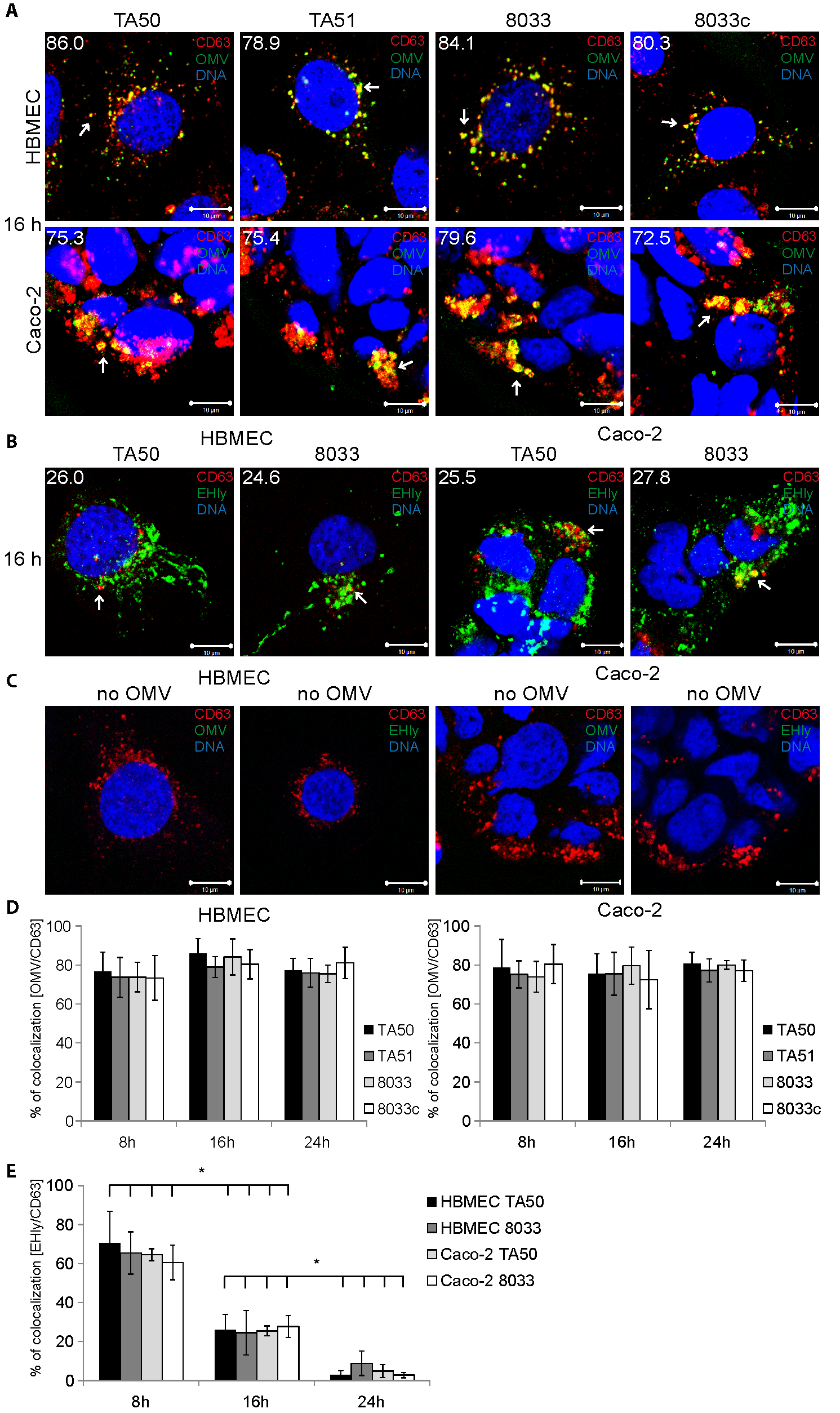

Supplement: Figure S5 — Colocalization of OMVs and EHEC-Hly with endo-lysosomal compartments detected with anti-CD63 antibody. (A) HBMEC and Caco-2 cells were incubated with EHEC-Hly-containing (TA50 or 8033) or EHEC-Hly-free (TA51 or 8033c) OMVs for 16 h. OMVs were stained with rabbit anti-E. coli LPS antibody and Alexa Fluor 488-conjugated goat anti-rabbit IgG (green), lysosomes with mouse anti-CD63 antibody and Cy3-conjugated goat anti-mouse IgG (red), and nuclei with DRAQ5 (blue). (B) HBMEC and Caco-2 cells were incubated with TA50 or 8033 OMVs for 16 h and stained as described above except that in lieu of OMVs, EHEC-Hly (EHly) was detected with rabbit anti-EHEC-Hly antibody and Alexa Fluor 488-conjugated goat anti-rabbit IgG (green). (C) HBMEC and Caco-2 cells were incubated for 24 h with 20 mM TRIS-HCl (OMV buffer) instead of OMVs and stained for OMVs or EHEC-Hly as described in (A) and (B). Pictures were taken using a laser-scanning microscope (LSM 510 META microscope, equipped with a Plan-Apochromat 63x/1.4 oil immersion objective). All three fluorescence images were merged and consisted of one optical section of a z-series with a pinhole of 1 airy unit. Colocalized red and green signals appear in yellow (examples are depicted by arrows). Scale bars are 10 µm. The percentages of colocalizations between OMVs and CD63-positive compartments (A) and EHEC-Hly and CD63-positive compartments (B) were calculated using BioImageXD6 colocalization tool and are indicated (averages from at least five different samples) by white numbers in the respective panels. (D, E) Graphical presentation of colocalizations between OMVs and CD63-positive compartments (D) and EHEC-Hly and CD63-positive compartments (E) during time (8 h, 16 h, 24 h) calculated using BioImageXD6 colocalization tool. * Significant differences between incubation times (p<0.05; paired Student's t-test). (TIF) [file ppat.1003797.s005.tif]

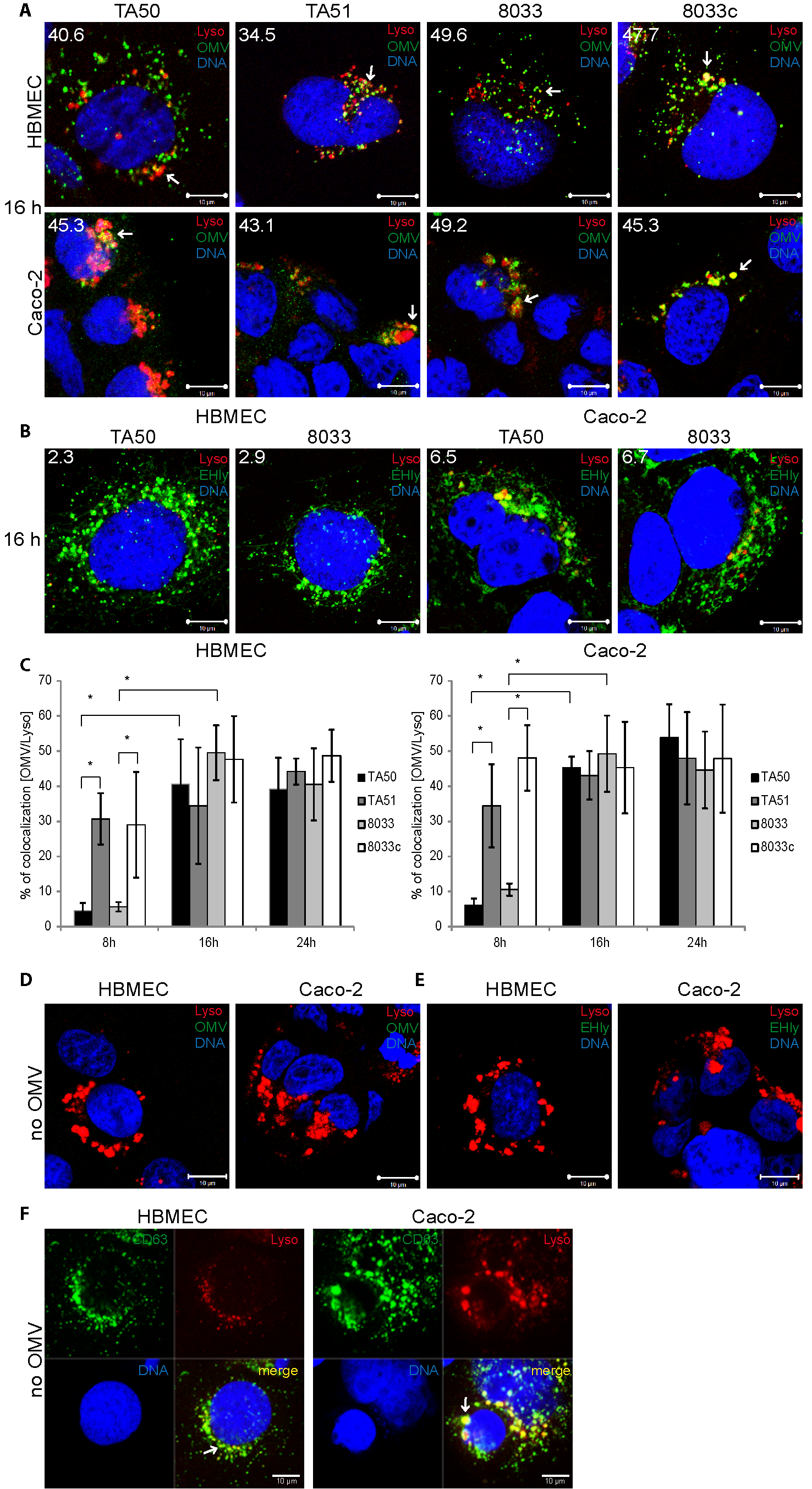

Supplement: Figure S6 — Releasing of EHEC-Hly from lysosomes leads to a transient loss of lysosomal function. (A) HBMEC and Caco-2 cells were incubated with EHEC-Hly-containing (TA50 or 8033) or EHEC-Hly-free (TA51 or 8033c) OMVs for 16 h. OMVs were stained with rabbit anti-E. coli LPS antibody and Alexa Fluor 488-conjugated goat anti-rabbit IgG (green), lysosomes with Lysotracker Red DND-99 (Lyso) (red) and nuclei with DRAQ5 (blue). (B) HBMEC and Caco-2 cells were incubated with TA50 or 8033 OMVs for 16 h and stained as described above, except that in lieu of OMVs, EHEC-Hly (EHly) was detected with rabbit anti-EHEC-Hly antibody and Alexa Fluor 488-conjugated goat anti-rabbit IgG (green). Pictures were taken and processed as described in the legend to Figure S5. Colocalized red and green signals appear in yellow (examples depicted by arrows). The percentages of colocalizations between OMVs and Lysotracker Red DND-99 (A) and EHEC-Hly and Lysotracker Red DND-99 (B) were calculated using BioImageXD6 colocalization tool and are indicated (averages from at least five different samples) by white numbers. (C) Graphical presentation of colocalizations between OMVs and Lysotracker Red DND-99 during time calculated using BioImageXD6 colocalization tool. * Significant differences between OMVs/incubation times (p<0.05; paired Student's t-test). (D, E) HBMEC and Caco-2 cells were treated with OMV buffer instead of OMVs and stained for OMVs (D) or EHEC-Hly (E) and lysosomes as described in (A) and (B). (F) Lysosomes in control, OMV-untreated cells were double-stained using anti-CD63 antibody and Alexa Fluor 488-conjugated goat anti-mouse IgG (green) and Lysotracker Red DND-99 (red); nuclei were stained with DRAQ5 (blue). Single channels and their merge are shown; colocalized red and green signals appear in yellow (examples depicted by arrows). Scale bars in all panels are 10 µm. (TIF) [file ppat.1003797.s006.tif]

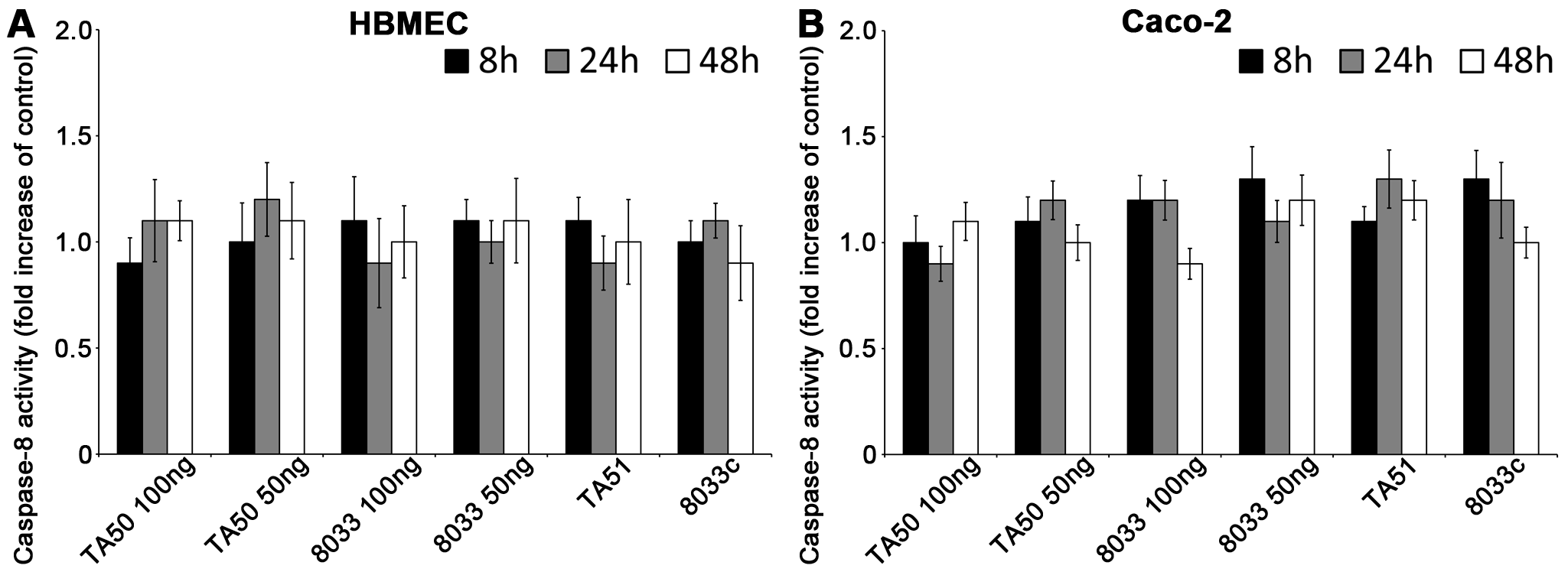

Supplement: Figure S7 — EHEC-Hly does not activate caspase-8. HBMEC (A) and Caco-2 cells (B) were incubated with EHEC-Hly-containing OMVs from strains TA50 or 8033 (20 µg or 10 µg of OMV protein containing 100 ng or 50 ng of EHEC-Hly, respectively) or with EHEC-Hly-free OMVs from strains TA51 or 8033c (20 µg of OMV protein) for the times indicated or remained untreated. Cells were lysed, the lysates were incubated with the colorimetric substrate of caspase-8 (Ac-IETD-pNA) and the color intensity, which is proportional to the level of caspase-8 enzymatic activity, was measured spectrophotometrically. The caspase-8 activity in OMV-treated cells was expressed as a fold-increase of that in untreated control cells (defined as 1). Data are means ± standard deviations from three independent experiments. (TIF) [file ppat.1003797.s007.tif]
