# Supplementary material for: Enterohemorrhagic Escherichia coli Hemolysin Employs Outer Membrane Vesicles to Target Mitochondria and Cause Endothelial and Epithelial Apoptosis
Source: PLoS Pathog. 2013 Dec 12;9(12):e1003797. doi: 10.1371/journal.ppat.1003797 (PMC3861543; doi:10.1371/journal.ppat.1003797)
Supplement: Table S1 — Presence of DNA in OMVs. (DOC) [file ppat.1003797.s008.doc]

Table S1. DNA content and presence of EHEC-Hly-encoding gene in EHEC-Hly-containing OMVs

| Strain | DNA amount (ng per 20 µg of OMV protein)^a^ in OMVs processed as indicated | | | EHEC-*hlyA*^b^ |
| --- | --- | --- | --- | --- |
|  | Intact  untreated | Intact  DNase-treated | Lysed after DNase treatment |  |
| TA50 | 1.4 ± 0.4 | 1.3 ± 0.5 | 2.4 ± 0.6 | - |
| 8033 | 1.2 ± 0.2 | 1.0 ± 0.3 | 1.9 ± 0.7 | - |

^a^ The protein amounts are expressed as averages ± standard deviations from three independent experiments.

^b^ Presence of EHEC-*hlyA* encoding EHEC-Hly was tested using PCR with primers hlyA1 and hlyA4 [7]; -, no amplicon obtained.
