# Supplementary material for: Enterohemorrhagic Escherichia coli Hemolysin Employs Outer Membrane Vesicles to Target Mitochondria and Cause Endothelial and Epithelial Apoptosis
Source: PLoS Pathog. 2013 Dec 12;9(12):e1003797. doi: 10.1371/journal.ppat.1003797 (PMC3861543; doi:10.1371/journal.ppat.1003797)
Supplement: Table S2 — Percentages of TA50, 8033, TA51 and 8033c OMVs internalized over time by HBMEC and Caco-2 cells. (DOC) [file ppat.1003797.s009.doc]

Table S2. Percentages of TA50, 8033, TA51 and 8033c OMVs internalized over time by HBMEC and Caco-2 cells

| Time (min) | % of internalized OMVs HBMEC^a^ | | | | % of internalized OMVs Caco-2^a^ | | | |
| --- | --- | --- | --- | --- | --- | --- | --- | --- |
|  | TA50 | TA51 | 8033 | 8033c | TA50 | TA51 | 8033 | 8033c |
| 5 | 33.3 | 43.9 | 17.7 | 0 | 31.8 | 44.6 | 24.0 | 29.5 |
| 15 | 35.7 | 41.6 | 15.8 | 34.4 | 28.4 | 43.9 | 32.5 | 38.5 |
| 30 | 32.7 | 39.4 | 26.1 | 22.4 | 29.8 | 29.2 | 47.1 | 64.9 |
| 60 | 48.6 | 45.6 | 64.9 | 74.4 | 31.4 | 79.1 | 47.4 | 41.0 |
| 120 | 63.2 | 58.9 | 81.0 | 61.0 | 42.8 | 41.7 | 45.7 | 52.9 |
| 180 | 70.9 | 86.8 | 82.6 | 84.1 | 43.6 | 49.7 | 55.2 | 51.5 |
| 240 | 73.6 | 79.7 | 84.4 | 100.0 | 48.1 | 50.8 | 72.0 | 56.6 |

^a^The percentage of internalized OMVs of each strain was calculated as the percentage of the fluorescence intensity of cells after quenching with trypan blue from the total fluorescence intensity of cells without trypan blue (means from three independent experiments).
